# Investigating the potential psychological significance of the alpha parameter in the Lévy flight model of decision making: A reliability analysis approach

**DOI:** 10.3758/s13428-025-02784-2

**Published:** 2025-08-26

**Authors:** Mehdi Ebrahimi Mehr, Jamal Amani Rad

**Affiliations:** 1 Institute for Cognitive and Brain Sciences, Shahid Beheshti University, Tehran, Iran; 2 Choice Modelling Centre & Institute for Transport Studies, University of Leeds, Leeds LS2 9JT, UK

**Keywords:** Lévy flights, Diffusion model, Decision-making, Test–retest reliability, Response time, BayesFlow

## Abstract

This study critically examines the cognitive and theoretical foundations of the alpha parameter within the Lévy flight model (LFM), an extension of the diffusion decision model (DDM) that incorporates heavy-tailed noise distributions. The alpha parameter, which modulates the tail of these distributions, is assessed for its test–retest reliability – an essential criterion for its classification as a cognitive style measure. Utilizing data from three previous studies, we observed that alpha demonstrates consistent reliability across tasks and time points, supporting its role as a trait-like characteristic. Our observation regarding the interrelations between LFM parameters showed that although most parameters exhibited weak correlations, reflecting their representation of distinct aspects of data, moderate correlations were observed between alpha and both threshold and non-decision time. Furthermore, investigating practice effects, we observed consistent reductions in non-decision time, threshold, and often alpha across sessions, accompanied by a corresponding increase in drift rate in demanding tasks. Notably, alpha showed a strong relationship with the mean reaction time of error responses, indicating its critical role in explaining fast error responses. Additionally, our examination of the predicted decision-time distribution found that lower alpha values correspond to shorter response times in the first quartile of both correct and error responses, highlighting its impact on capturing the dynamics of fast decision-making. Employing the BayesFlow framework for parameter estimation, we evaluated its precision across varying trial counts. These findings offer insights for future research on LFM and similar models.

**Supplementary Information:**

The online version contains supplementary material available at 10.3758/s13428-025-02784-2.

“*The new science addresses seemingly straightforward questions like these:*

- *How effective is a given treatment in preventing a disease?*
- *Did the new tax law cause our sales to go up, or was it our advertising campaign?*
- *What is the health-care cost attributable to obesity?*
- *Can hiring records prove an employer is guilty of a policy of sex discrimination?*
- *I’m about to quit my job. Should I?*”

— (Pearl & Mackenzie, 2018)

*In line with the fifth fundamental causal inquiry – “I’m about to quit my job. Should I?” – posed by* Pearl and Mackenzie (2018), *this paper examines an analogous question within decision-making research: We are about to invest into studying the Lévy flight model. Should we?*

## Introduction

Cognitive scientists are curious to understand how the mind processes information to make decisions, and computational models play a key role in revealing these mechanisms. Sequential sampling models (SSMs) are particularly effective for examining how humans gradually accumulate evidence to reach a decision (Ratcliff & Smith, 2004). Among SSMs, the diffusion decision model (Ratcliff, 1978) is the most prominent framework for binary response time tasks, due to its fit across various cognitive tasks and the validity of its parameters in multiple paradigms (Arnold et al., 2015; Lerche & Voss, 2017). DDM assumes that decision-making is based on continuous evidence accumulation described by a Wiener diffusion process. In this model, noisy information accumulates over time from a starting point (*zr*) toward one of two decision boundaries (*a* or 0). The drift rate (*v*) indicates the speed of information accumulation, while non-decision time (*ndt*) represents both stimulus encoding and response execution. DDM uses simplified assumptions due to parameter estimation constraints. However, advanced methods like BayesFlow (Radev et al., 2020) have mitigated these limitations, paving the way for the development of more complex models. One of the simplified assumptions in the DDM is the distribution of the noise. DDM assume that the noise is normally distributed which in its core holds the finite variance assumption. LFM emerges when the finite variance assumption in the noise of the DDM is relaxed, resulting in a model that incorporates a stable distribution.

Lévy flights, popularized by Mandelbrot (1982), are characterized by random walks with heavy-tailed distributions that allow for infrequent but significant jumps. As discussed by Nolan (2020), these models have been applied across various physical and biological contexts, where traditional Gaussian models fall short in explanation, including radio wave propagation in space (Boldyrev & Gwinn, 2003; Freeman & Chisham, 2005), animal foraging (Viswanathan et al., 1999), human travel (Brockmann et al., 2006), and the spread of epidemics (Boto & Stollenwerk, 209; Brockmann & Hufnagel, 2007; Linder et al., 2008; Machado & Lopes, 2020). In cognitive science, Lévy flights have been used to model aspects of human cognition, such as memory retrieval (Rhodes & Turvey, 2007) and decision-making (Patten et al., 2020; Stephen et al., 2009).

While these applications mostly focus on the geometry of the paths themselves, in modeling human decision-making, the interest must shift from specific trajectories to the holistic outcomes these processes produce – specifically, the distribution of reaction times (RT) in decision tasks.

Building on this perspective, in Section “Lévy flight model and noise”, we present our conceptualization of the Lévy flight model (LFM), outlining both its theoretical motivations and its particular appeal for modeling reaction time. We also briefly review the origin and development of the model, particularly in the context of evidence accumulation models in decision-making, as discussed in the following.

Voss et al. (2019) were the first to introduce the LFM as an extension of the DDM. To account for jumps in the decision process, they applied the LFM in the realm of evidence accumulation to model extreme events, similar to stock market crashes (Mantegna, 1991) or shifts in predator hunting zones (Viswanathan et al., 2008), which could also occur in human decisions. The LFM replaces the Gaussian noise assumption in the DDM with heavy-tailed noise, allowing for infrequent but extreme events that manifest as jumps in the decision process.

Voss et al. (2019) demonstrated that, based on AIC, the full LFM – accounting for inter-trial variability – provided the best fit in both the single-stimulus task and the multi-stimulus task (under accuracy-focused instruction). However, when using BIC, which penalizes complexity more heavily, simpler versions of the LFM were favored, particularly for easier tasks and speed-focused conditions.

**Fig. 1:**
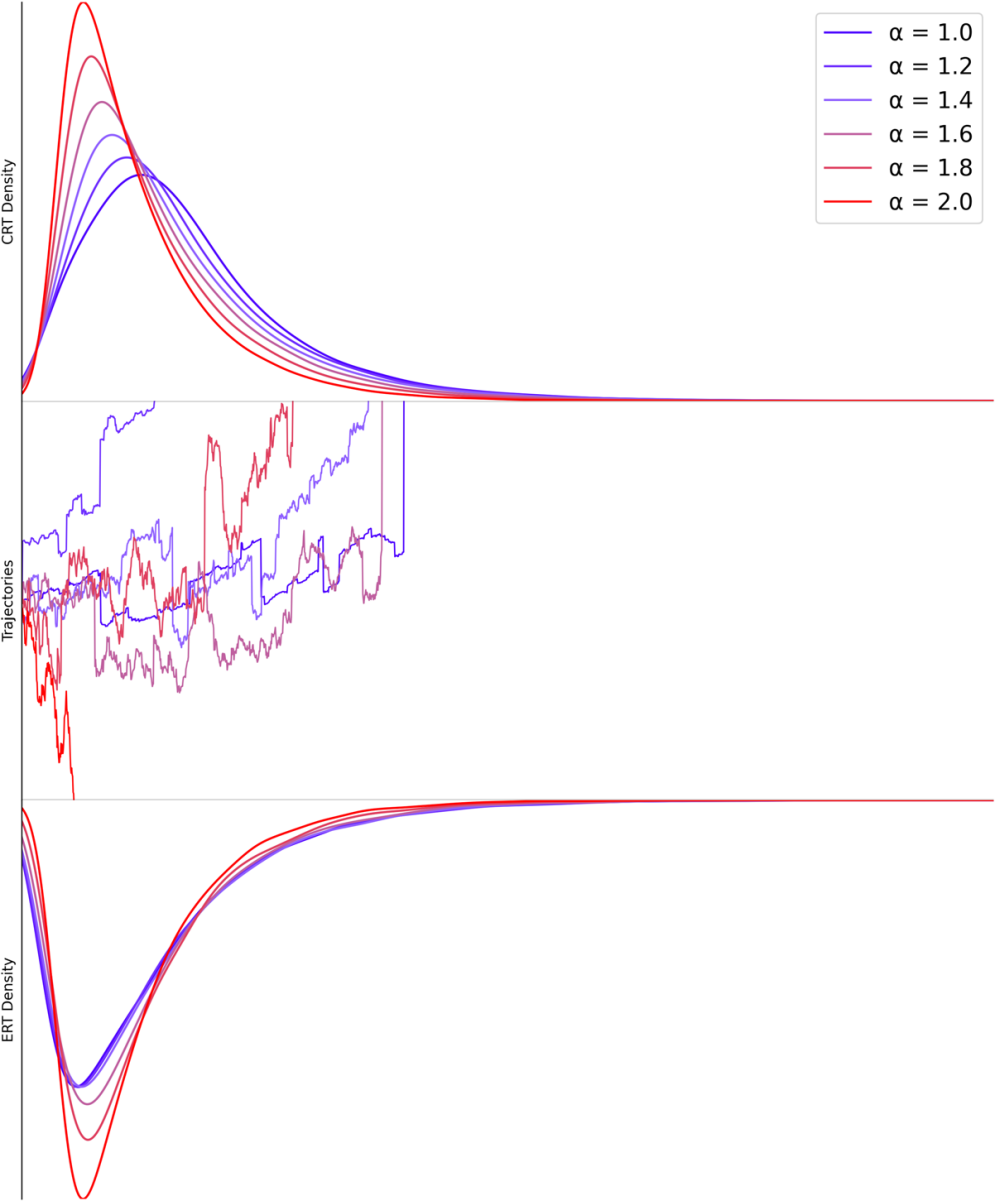
Predicted decision-time distributions generated by LFM. The *upper panel* displays correct response times (CRT), while the *lower panel* shows error response times (ERT), both generated using six distinct $\alpha$-stable noise distributions. The *middle panel* presents sample LFM trajectories across varying $\alpha$ values, illustrating the effect of $\alpha$ on a decision process. When $\alpha = 2$, the noise is Gaussian, producing smooth trajectories. As $\alpha$ decreases below 2, the noise distribution exhibits infinite variance, resulting in more frequent and pronounced jumps. At $\alpha = 1$, the distribution becomes Cauchy, leading to highly irregular dynamics. Further details are discussed in the main text

In the LFM, the probability of extreme events is regulated by the stability parameter $\alpha$, where the Gaussian and Cauchy distributions, characterized by $\alpha = 2.0$ and $\alpha = 1.0$ respectively, are special cases of stable distributions (see Fig. 1).

A significant problem with LFM is its lack of a firm theoretical framework, which leaves the stability parameter ($\alpha$) without intrinsic meaning unless such a framework is established. This issue parallels that of the ex-Gaussian distribution, which, although frequently used to summarize RT distributions, also lacks theoretical interpretation. As Yap et al. (2012) notes, citing Matzke and Wagenmakers (2009) and Schmiedek et al. (2007), ex-Gaussian parameters are useful for capturing empirical data but should not be directly linked to cognitive processes without a theoretical foundation. Mapping these parameters onto specific cognitive processes, as cautioned by Hohle (1965) and McGill and Gibbon (1965), is risky without a theoretical framework. In the same way, while the LFM effectively captures the heavy-tailed nature of decision-making in response to extreme events, $\alpha$ remains a descriptive parameter without cognitive interpretation. Accordingly, model fit provides a measure of alignment between data and the assumptions embedded in the model. However, such a fit should not be conflated with confirmation of ontological accuracy. High fit does not necessarily imply that the model reflects the true “generative process”, only that it captures observable patterns under a given formal framework.

The primary aim of this study is to address this limitation by exploring the potential psychological significance of the stability parameter ($\alpha$), emphasizing that its test–retest reliability is essential for classifying it as a cognitive style measure (Lerche & Voss, 2017; Schubert et al., 2016).

Although studies investigating ($\alpha$) are limited, Wieschen et al. (2020) found that the complex LFM produce lower BIC values than its diffusion model counterpart in both tasks in their study and more notably, reported correlations in $\alpha$ across tasks. This finding (the observed correlation) evoked interest in whether alpha measures stable inter-individual differences in decision-making. They proposed two opposing hypotheses to explain its theoretical basis, aiming to guide future studies by incorporating other measures of cognitive performance, such as intelligence, working memory, or cognitive flexibility for testing.

We were interested in whether individual differences in response to unpredictable factors (henceforth referred to as “latent perturbations”) could be reflected in the variation captured by the alpha parameter. However, rather than directly exploring these factors, our “initial” step focused on assessing the stability of $\alpha$ itself. Establishing reliability is essential, as it ensures that the parameter can consistently capture meaningful variation. If $\alpha$ reflects a trait-like signature in how individuals adapt to extreme variance, its stable measurement may open new doors for interpreting decision making beyond central tendency models. Consequently, this evidence suggests that future studies on the LFM or “similar” models are worthwhile.

Using data from three previous studies with 12 decision-making tasks (Lerche & Voss, 2017; Schubert et al., 2016; Yap et al., 2012), we conducted a comprehensive analysis of LFM parameters.

We assessed test–retest reliability coefficients to evaluate the stability of the LFM parameters. Furthermore, we computed the mutual information (MI) between parameters across sessions to quantify shared information and capture non-linear reliability. Additionally, we examined correlations among LFM parameters to investigate whether they represent distinct aspects of the data. Using gradient boosting regression (GBR; Friedman (2001); Pedregosa et al. (2011)), we investigated the intrinsic reliability of the alpha parameter. To further assess the portion of reliability uniquely attributable to alpha, we employed conditional mutual information (CMI; Cover and Thomas (2006)) analysis. Additionally, through regression models, we explored specific data aspects captured by alpha, such as its relationship with mean reaction times of error responses. Furthermore, we analyzed the predicted decision-time distribution characteristics concerning alpha. We also examined the impact of practice on information processing and performance.

Key findings from our study confirmed alpha’s reliability across two sessions in all tasks, while significant across-task reliability was observed in the more demanding tasks. Comparisons between theoretical MI and correlation plots revealed that mutual information captures more shared information than linear correlation alone. Our analysis of the interrelations between LFM parameters revealed that, although most parameters showed weak correlations – underscoring their representation of distinct aspects of the data – moderate correlations were observed between alpha, threshold, and non-decision time. GBR and CMI analyses confirmed that alpha’s reliability is inherent and not inherited from other parameters. We also found that alpha has a strong relationship with mean error reaction time, indicating its critical role in explaining fast error responses. Additionally, our examination of the predicted decision-time distribution found that lower alpha values correspond to shorter response times in the initial quartile of both correct and error responses. Practice effects from session one to session two improved information processing and performance, as evidenced by increased drift rates and reduced non-decision times and thresholds, which were associated with lower alpha values.

These results highlight alpha’s potential psychological significance in capturing individual differences related to cognitive functioning, emphasizing the need for a theoretical framework to fully interpret these findings. Future research should focus on developing this framework and expanding empirical studies to explore the implications of alpha in cognitive processing across various decision-making paradigms.

In the following sections, we first introduce the LFM and the role of noise within the model. Subsequently, we have a section dedicated to “reliability”, the concept we employed to examine whether the alpha parameter in the LFM is eligible to have psychological significance. We will then proceed to describe the data and report the findings, followed by a discussion of our results and a concluding section.

## Lévy flight model and noise

Human decision-making is inherently uncertain, shaped by a range of unpredictable internal and external influences. In modeling terms, these influences – ranging from fluctuations in sensory input and transient cognitive states to contextual and environmental noise – can collectively be referred to as latent perturbations (LPs). These perturbations cannot be directly observed or precisely predicted, yet they significantly impact the randomness of the decision-making process.

In the diffusion decision model, the LPs are modeled as normally distributed noise, which assumes finite variance. This assumption is rooted in the classical central limit theorem, which states that the normalized sum of independent, identically distributed variables with finite variance converges to a normal distribution. However, when the assumption of finite variance is relaxed, the generalized central limit theorem posits that the only possible limiting distributions are stable distributions (Nolan, 2020). Accordingly, by dropping the finite-variance assumption in the DDM, we arrive at a model governed by a stable distribution, commonly known as the Lévy flight model (Voss et al., 2019).

Crucially, the DDM assumes, rather than empirically verifies, the suitability of modeling LPs with a normal distribution. This assumption is ad hoc: a modeling assumption introduced to make the model “work”, often without independent justification. However, the assumption may not hold in real-world cognitive processes, where rare but impactful events can drive behavior. The LFM, through its use of a stable distribution, allows for infrequent but large deviations from the drift rate, called ‘jumps’, in the decision processes (Voss et al., 2019). The probability of such extreme events is governed by an additional parameter, alpha, which determines the heaviness of the tails in the noise distribution.

The key distinction between the LFM and the DDM lies in this additional parameter. When treated as a free parameter, alpha enables the model to capture individual differences in sensitivity to LPs. In this framework, alpha potentially encodes meaningful psychological information. The jumps result in reaction time distributions with distinctive characteristics (see Fig. 1). Specifically, the heavy tails of the stable distribution imply a non-negligible probability of extremely fast or slow responses far from the mean – outcomes that would be exceedingly rare under the DDM. Consequently, LFM-generated reaction time distributions typically exhibit larger standard deviations and steeper lower edges compared to those modeled by DDM.

Such patterns suggest that the LFM accommodates a broader and more realistic range of behavioral outcomes, particularly in contexts where LPs may exert rare but substantial effects. This perspective frames the alpha parameter not merely as a technical refinement but potentially as a theoretically and psychologically significant dimension, one that may capture individual differences in sensitivity to LPs. As a first step, if alpha proves to be reliable across time and task, it could serve as an important marker, helping to bridge computational modeling with cognitive theory. Therefore, we invite researchers to contribute to the development of a theoretical framework that explains the psychological relevance of alpha, and to explore its empirical implications.

Mathematically, the process of information accumulation in the LFM can be represented as follows (Voss et al., 2019):

$$ \left\{ \begin{aligned} X(0)&= zr, \\ X(t \!+\! \Delta t) \!&= \! X(t) + v \cdot \Delta t + e \Delta t^{\frac{1}{\alpha }}, \!\!\! \quad e \sim \text {Stable}(\alpha , \beta \!=\! 0, \\ \gamma&= 1, \delta = 0), \quad 1< \alpha < 2, \end{aligned} \right. $$

where $X(t)$ represents the accumulated evidence at time $t$, starting from $X(0) = zr$, where $zr$ is the initial starting point of the decision process, often reflecting a “bias” toward one decision boundary or the other. The process continues until the state variable $X(t)$ reaches or exceeds an upper threshold $a$, or falls to zero, at which point the process terminates. The variable $t$ represents the time elapsed during the decision-making process, while $v$ denotes the drift rate, which is the average rate of evidence accumulation over time. The time step $\Delta t$ represents discrete intervals in the decision process. After the decision process concludes, a non-decision time *(ndt)*, which accounts for stimulus encoding and motor execution, is added to the total time derived from this formula.

The term $\text {Stable}(\alpha , \beta = 0, \gamma = 1, \delta = 0)$ refers to a stable distribution characterized by four parameters. The parameter $\alpha$ controls the heaviness of the distribution’s tails, influencing the likelihood of extreme values occurring. The parameter $\beta$, set to zero, defines the skewness of the distribution; a value of zero ensures the distribution remains symmetric. The parameter $\gamma$, set to one, serves as the scale parameter, determining the spread or width of the distribution. The parameter $\delta$, set to zero, defines the location of the distribution, centering it at zero (Nolan, 2020; Nikias & Shao, 1995; Samorodnitsky & Taqqu, 1995; Zolotarev, 1986).

Voss et al. (2019) were the first to introduce the LFM as an extension of the DDM to account for jumps in the decision process. Subsequent studies have employed the LFM for specific inquiries within the field (Rasanan et al., 2021; Wieschen et al., 2023, 2020). Wieschen et al. (2020) explored the significance of the alpha parameter, while Wieschen et al. (2023) utilized the LFM to examine age-related differences in decision-making processes. Schumacher et al. (2023) provided the first experimental validation of superstatistics and conducted a formal comparison of four non-stationary DDM within a specially designed perceptual decision-making task. The current study aims to examine the reliability of the LFM parameters to provide evidence for a potential theoretical framework that allows for ‘jumps’ in the decision-making process, thereby justifying further investigation.

There is a subtle distinction between the concept of reliability in psychological testing and its application in computational models. We will discuss this distinction and outline the guiding logic of our research, which is based on a reliability analysis, in the following section before proceeding with the data description.

## Reliability in computational models

In the field of psychology, the concepts of reliability and validity are essential for determining the accuracy with which a test measures the ‘constructs’ it is supposed to assess (American Educational Research Association et al., 2014). It is important to demonstrate the reliability of these tests to support their interpretation and use, based on the expectation that the performance of individuals or groups will be consistent across different testing administrations (American Educational Research Association et al., 2014). Following this premise in psychological testing, computational models like the DDM, with parameters such as drift rate and threshold, do not aim to assign a numerical value (score) to a “**construct**”. Instead, these models aim to simplify complex behavioral data into a simpler, testable model, and the parameters of these models could represent different constructs in different tasks that could be modeled by DDM.

These models and their parameters, serving as reflections of, or ‘windows into,’ cognitive and/or neural processes, segment these processes into identifiable, unique components (Eckstein et al., 2021). This segmentation enables the assessment of individuals’ inherent characteristics (Eckstein et al., 2021). Therefore, just as in psychological assessments, the parameters of computational models must exhibit a form of reliability to ensure they meaningfully reflect the process components they are designed to represent. The critical distinction is that reliability in psychological testing depends on the assumption that the ‘constructs’ being measured are stable attributes of individuals, necessitating consistent scores. On the other hand, computational models concentrate on the dynamic nature of cognitive processes, rather than solely on a specific trait.

In psychological research, several types of reliability are commonly evaluated: test–retest reliability checks the consistency of a test over time (Crocker & Algina, 1986); inter-rater reliability evaluates how different observers rate the same phenomenon (McHugh, 2012); parallel-forms reliability compares the scores from two versions of a test to ensure they measure the same construct equivalently (Nunnally & Bernstein, 1994); internal consistency reliability often gauged by Cronbach’s alpha, assesses the coherence among test items to ensure they collectively measure a single construct effectively (Cronbach, 1951), and split-half reliability divides the test to check consistency within itself (Spearman, 1910).

Previous research on the reliability of the computational models is scarce, mostly because of the aforementioned difference between the index of psychological construct and computational model parameters, the reliability analysis for the DDM was not of primary interest, and its importance was only recently recognized. In the realm of decision-making, some research has been conducted through the lens of “reliability” with subtle differences in their motivation and evaluation. However, they share a common concern regarding the DDM parameters’ ability to detect individual differences reliably. For instance, Yap et al. (2012) examined differences among individuals in word recognition performance. They assessed between-session reliability (test–retest reliability) along with within-session reliability (split-half reliability) of ex-Gaussian parameters and diffusion decision model parameters. Within-session reliability was impressively high and between-session reliability assessment of the main parameters of the diffusion model indicated a relatively high correlation ($0.645< r < 0.736$). The findings provide evidence that readers bring with them a specific RT distributional “signature” that goes beyond the simple mean performance. Lerche and Voss (2017) evaluated the retest reliability of the diffusion model parameters in two studies, each with two sessions apart. They also found satisfying test–retest reliability of the main parameters ($r > 0.7$ for drift and threshold ). They also report the across-task correlation (parallel-forms reliability) of the parameters. This research also investigated the influence of the number of trials on the retest reliability in a third study. Schubert et al. (2016) conducted a latent state-trait analysis of diffusion model parameters to assess their trait characteristics in a study within two sessions 8 months apart (the previous two studies we mentioned were 1 week apart). Elementary Cognitive Tasks (ECTs) were chosen because they are tasks with minimal cognitive demands that minimize unwanted sources of variance such as strategy use and ’learning effects’. They found consistency coefficients to be low to moderate, except for the drift rate, which was high.

In this study, we are aware that the introduction of the LFM into the decision-making field was more based on its better fit to the data (Voss et al., 2019), rather than a firm theoretical framework. With this in mind, we proceed under the “assumption”^1^ that such a theoretical framework exists, thus, the interpretability of its parameters depends on their reliability. Our goal is to examine the reliability of the LFM parameters to provide evidence supporting the existence of a potential theoretical framework that accounts for ‘jumps’ in the decision-making process, thereby justifying further investigation. In addition, we conducted additional analyses to direct future studies by deriving insights from the results. To this end, we reanalyzed the dataset from three previous studies (Lerche & Voss, 2017; Schubert et al., 2016; Yap et al., 2012), applying the LFM to the data. Before we detail the data description and results in greater depth, we will first briefly discuss one of the key factors that significantly influence reliability “measures”.

*More trials increase reliability*: Reliability measures can be significantly affected by various factors, which, if overlooked, may lead to incorrect interpretations of results. It is important to note that any assessment of accuracy or reliability is specific to a particular assessment procedure and may change if significant alterations are made. In general, reliability tends to increase if the assessment is expanded to include more comparable tasks or items, and it tends to decrease if the assessment is shortened, such as by reducing the number of items or tasks. Therefore, a common and effective strategy to enhance reliability is to lengthen the assessment, thereby increasing the size of the sample of tasks/items, raters, or occasions involved (American Educational Research Association et al., 2014). This is because increasing the number of trials reduces the impact of between-trial variations, allowing us to capture the inherent scores more accurately. Lerche and Voss (2017) demonstrated in their third study that the reliability of the diffusion model increases with the growth in the number of trials. Furthermore, the high reliability coefficients reported in Yap et al. (2012), which involved 2000 trials, further support the idea that the number of trials similarly affects the measure of reliability coefficients.

In the following, after describing the dataset, we will present the reliability coefficients (i.e., between-session and across-tasks reliability) for the LFM parameters for each task and for both models we trained. Then, we will proceed to the five analyses discussed in the introduction.

## Data description

To explore the existence of a theoretical framework that accounts for jumps in the decision process and to assess the potential psychological significance of the alpha parameter through test–retest reliability analysis, we sought datasets collected over two sessions and across different tasks. Our search revealed a paucity of research addressing the reliability of diffusion model parameters. Ultimately, we secured data from three studies. The first dataset originates from the study by Lerche and Voss (2017). The second dataset was provided by Yap et al. (2012), and the third dataset stems from investigation of diffusion model parameter consistency by Schubert et al. (2016). In the following, we will succinctly present each dataset. Since the original articles provide a detailed overview of the methodological aspects of the data, our focus will be on highlighting the most significant elements of the databases.

### Dataset 1: Lerche and Voss (2017)

Lerche’s dataset (Lerche & Voss, 2017) encompasses two studies. In the first, 105 participants engaged in both the Lexical Decision Task (LDT) and the Recognition Memory Task (RMT) over two sessions, spaced a week apart. The RMT utilized picture stimuli, thereby contrasting the LDT’s word-based tasks with those based on images (LDT: words vs. RMT: pictures). The LDT involved 200 German nouns, each with one or two syllables and 4–6 letters, and for each noun, a corresponding non-word was generated through random vowel substitution.

For the RMT, 200 images from the International Affective Picture System (IAPS; Lang et al. (2008)) and the Emotional Picture Set (Wessa et al., 2010), characterized by neutral valence (4-–6 on a 1–9 scale) and low arousal (up to 5 on a 1–9 scale), were selected. Only pictures showing humans were selected to have a relatively homogeneous set of stimuli, thus making the task more “demanding”. The order of tasks (LDT and RMT) was counterbalanced across participants.

In the second study, aimed at generalizing the findings to a different experimental paradigm, 114 participants performed an associative priming task (APT) in two sessions 1 week apart. This task, based on a study by Voss et al. (2013), involved a Lexical Decision Task where targets (words or non-words) were preceded by primes associated or not associated with the target. Each session contains 400 prime-target pairs, with half of the primes closely associated with their targets, and the other half not.

For this dataset, we noticed during the final revision of preparing our manuscript, just before submission, that there is a related study by Wieschen et al., which was under review, focusing on reliability analyses of the LFM parameter using the Lerche and Voss (2017) dataset from another perspective. While both studies utilize this shared dataset, as will be evident in the following, our work integrates several other large datasets, and Wieschen et al. have employed additional data in their analyses. These distinct datasets and perspectives complement each other, contributing to a more comprehensive understanding of the cognitive mechanisms underlying decision-making.

### Dataset 2: Yap et al. (2012)

The dataset utilized in Yap’s study (Yap et al., 2012) was based on archival trial-level data from the English Lexical Project (ELP). A total of 819 participants engaged in the Lexical Decision Task (hereafter referred to as “ELP” within this article) across two sessions, which were held no more than 1 week apart. All participants were native speakers of English. The first session had 2000 trials, and the second session had 1374 trials. Nonword trials on the task were legal nonwords that did not sound like real words.

### Dataset 3: Schubert et al. (2016)

Schubert’s dataset (Schubert et al., 2016) is different from the two datasets we introduced. The interval between its two sessions spanned 8 months, and elementary cognitive tasks (ECTs) were chosen because they are tasks with minimal cognitive demands that minimize unwanted sources of variance such as strategy use and learning effects. A total of 114 participants completed both sessions. There were three tasks in this study. In the Visual Choice Response Time Task, participants were presented with one (CR1), two (CR2), or four (CR4) response alternatives (we will also refer to these tasks with 0-bit, 1-bit and 2-bit). Each condition started with ten practice trials with immediate feedback and was followed by 200 test trials without feedback. During the Sternberg Memory Scanning Task, participants were shown a memory set consisting of one (S1), three (S3), or five (S5) digits, displayed on a black screen. Each condition was initiated with ten practice trials with instant feedback, followed by 100 test trials without feedback. The order of conditions was counterbalanced across participants. The Posner Letter Matching Task required participants to determine if two letters were identical. In the physical identity (PI) condition, the task was to assess if the letters were physically identical, while in the name identity (NI) condition, it was to decide if the two letters had the same name. The position of keys indicating whether the letters were identical was counterbalanced across participants. Each condition began with ten practice trials with immediate feedback followed by 300 test trials without feedback. All participants started with the PI condition at the first laboratory session, whereas all participants started with the NI condition at the second laboratory session.

Table 1 briefly outlines the tasks analyzed in this paper and the coding scheme applied throughout the article.

**Table 1:** Summary of data characteristics

Studies	NPartic	Task	Coded	Interval	Cond	Trs
Yap	819	Lexical Decision Task	ELP	1 Week	2	2000
Lerch Study 1	105	Lexical Decision Task	LDT	1 Week	2	400
Lerch Study 1	105	Recognition Memory Task	RMT	1 Week	2	200
Lerch Study 2	128	Associative Priming Task	APT	1 Week	4	400
Schubert	114	Visual Choice Response Time Task (CR1)	0-bit(ECTs)	8 Months	1	200
Schubert	114	Visual Choice Response Time Task (CR2)	1-bit(ECTs)	8 Months	1	200
Schubert	114	Visual Choice Response Time Task (CR4)	2-bit(ECTs)	8 Months	1	200
Schubert	114	Sternberg Memory Scanning Task (1 set)	S1(ECTs)	8 Months	1	100
Schubert	114	Sternberg Memory Scanning Task (3 set)	S3(ECTs)	8 Months	1	100
Schubert	114	Sternberg Memory Scanning Task (5 set)	S5(ECTs)	8 Months	1	100
Schubert	114	Posner Letter Matching Task (PI)	PI(ECTs)	8 Months	1	300
Schubert	114	Posner Letter Matching Task (NI)	NI(ECTs)	8 Months	1	300

This table summarizes the tasks analyzed in this study. The table includes information on the number of participants (NPartic), the type of task performed (Task), coded identifiers for each task (coded), the interval between sessions (Interval), the number of conditions (Cond), and the number of trials (Trs). Note: All datasets have an equal number of trials for both sessions, except for the Yap study, where session 2 had 1374 trials instead of the 2000 trials listed

## Parameter estimation

### Amortized Bayesian inference with BayesFlow

The LFM lacks a closed-form likelihood (Voss et al., 2019). In these situations, scientists often turn to simulation-based methods for estimating the parameters of their model (Radev et al., 2020). BayesFlow leverages Bayesian inference principles and normalizing flows to effectively learn a direct probabilistic relationship between data and parameters (Radev et al., 2020). This is achieved through a dual neural network architecture consisting of an invertible neural network and a summary network. These networks are trained using simulated data generated by the known forward model, incorporating appropriate priors. The priors for our study were selected based on Wagenmakers’ research (Wagenmakers et al., 2007)^2^ and the alpha parameter was constrained to the interval [1, 2]. BayesFlow utilizes amortized inference, where estimation is split into a potentially expensive upfront training phase, followed by a much cheaper inference phase. This approach enables quick and efficient application of learned networks to new data without the need for retraining from scratch. Bayesian inference of this method could be validated with simulation-based calibration (SBC) (Talts et al., 2018).

### Modeling LFM: Simulation and parameter inclusion

For our simulations, we followed a procedure similar to the one conducted by Lerche and Voss (2017) adding just the parameter alpha to the models. Thus in the Model 1 (M1) for LDT and RMT, we included the parameters boundary separation (*a*), starting point (*zr*), two drift rates for the two stimulus types (*v2* for non-words and new pictures associated with the lower threshold, *v1* for words and old pictures associated with the upper threshold), non-decision time (ndt), between trial variability of non-decision time (*sndt*), and the stability parameter alpha.

For Model 2 (M2) of these tasks, we fixed the starting point (*zr* = 0.5) and between trial variability of non-decision time (*sndt* = 0), thus including threshold separation (*a*), two drift rates for the two stimulus types (*v2* for non-words and new pictures associated with the lower threshold, *v1* for words and old pictures associated with the upper threshold), non-decision time (*ndt*), and the stability parameter alpha.

We did the same for the data from ELP. The only difference is the range of the number of trials (in this case, networks were trained for [100–2000] trials).

The parameter estimation process for APT closely mirrored that of LDT and RMT, with the primary distinction being the number of estimated parameters. Previously, we estimated two drift rates for LDT, one for each stimulus type (words vs. non-words). However, for APT, we estimated four drift rates and four non-decision times, tailored to the combinations of prime type (associated vs. non-associated) and target type (word vs. non-word). For data from Schubert, we were unable to train an accurate network including the starting point (*zr*) and variability in non-decision time (*sndt*) as free parameters to be estimated for such a small number of trials in the tasks. Moreover, considering that ECTs pose minimal cognitive demands, including variability for non-decision time do not align logically with our expectations of task performance, nor do we anticipate any bias to add the starting point (*zr*) as a free parameter to the model. Therefore, we have just one estimation network with parameters threshold separation (*a*), one drift rate, non-decision time (*ndt*), Alpha controlling the heaviness of the tail of the noise distribution in our analysis for this dataset. The starting point and variabilities are fixed (*zr* = 0.5, *sndt* = 0).

We trained all networks with a wide range of trial numbers for two primary reasons. First, due to the variability in trial numbers within the data, resulting from non-responses and the removal of outliers during data pre-treatment, and the variance in trial counts across different tasks, especially notable in the ELP data where session one contains 2000 trials compared to 1374 in session two. Second, to demonstrate the method’s accuracy across various trial numbers, providing a benchmark for its performance that can inform future study design. Figure A1 in the appendix shows the performance of the trained networks over all trial numbers. Plots of the simulation-based calibration for each network are also presented in Appendix Figure C3.

## Results

We conducted six analyses on the estimated parameters, with our main focus on assessing potential significance of the alpha parameter. Initially, we verified the reliability of the LFM parameters. With their reliability confirmed, we moved on to explore the practice effect between the first and second sessions through a *t* test analysis, aiming to gain further insights into the alpha parameter’s significance. In our third analysis, we examined whether the parameters of the LFM capture distinct aspects of the data. Consequently, the fourth analysis investigated whether the observed reliability of alpha is intrinsic or influenced by other parameters. We then explored “Which Aspect of Data is being Captured by Alpha”, using additional regression analyses, and concluded with an analysis of the features of the predicted decision-time distribution to understand the dynamics of fast decision-making. We will start by detailing the data pre-treatment process. The results of our analyses are presented in this section, and we plan to discuss our findings in the “Discussion” section.

### Data pre-processing

We followed the data pre-treatment protocols as established in the original studies (Lerche & Voss, 2017; Schubert et al., 2016; Yap et al., 2012). In Lerche’s dataset (Lerche & Voss, 2017), due to technical issues, data from one participant were missing from the RMT task in session two, and another participant’s data were excluded due to low accuracy scores. Consequently, we analyzed data from 104 participants for the LDT and 103 for the RMT. Reaction times below 200 ms or above 2500 ms were excluded for both tasks. Similarly, in the APT task, trials with RTs outside this range were excluded.

From Yap’s data (ELP) (Yap et al., 2012), we utilized data from 805 participants, excluding trials where response latencies were faster than 200 ms or slower than 3000 ms. Additionally, RTs exceeding 2.5 standard deviations from each participant’s mean were identified as outliers.

Schubert’s study (Schubert et al., 2016) discarded any RTs faster than 100 ms or slower than 3000 ms, and in the second step, any trials with log-transformed RTs exceeding ± 3 standard deviations of each condition mean at the intra-individual level were excluded. Following this approach, we applied the same criteria in our analysis.

To ensure that variables were almost normally distributed – a requirement for calculating Pearson correlation coefficients – we applied transformations to some variables. For the alpha parameter, values were first subtracted by 1, followed by an arcsin transformation and then a Box-Cox transformation. A similar procedure was used for accuracy rates but without the subtraction step. The mean of the log-transformed RTs for correct responses (mean RT) was computed as the representative measure going through the analysis. We assumed that the remaining parameters followed a normal distribution. Plots illustrating the distributions of these parameters can be found in the Appendix, specifically in the section titled *Correlation Among LFM Parameters* (see Appendix E).”

**Fig. 2:**
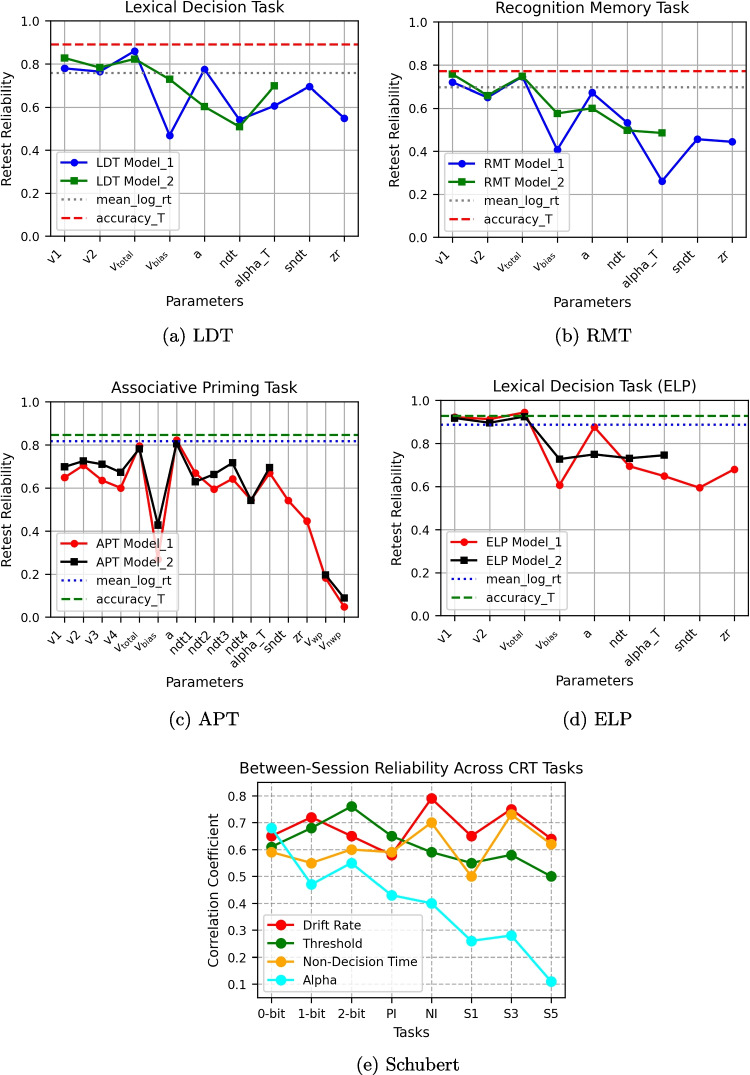
Retest reliability of LFM parameters across different tasks and models: **a** Lexical Decision Task (LDT), **b** Recognition Memory Task (RMT), **c** Associative Priming Task (APT), **d** Lexical Decision Task from the English Lexical Project (ELP), and **e** Elementary Cognitive Tasks from Schubert’s study. Each panel compares the reliability coefficients of Model 1 and Model 2, for the LFM parameters, along with the retest reliability of the transformed accuracy rate and mean reaction time

**Fig. 3:**
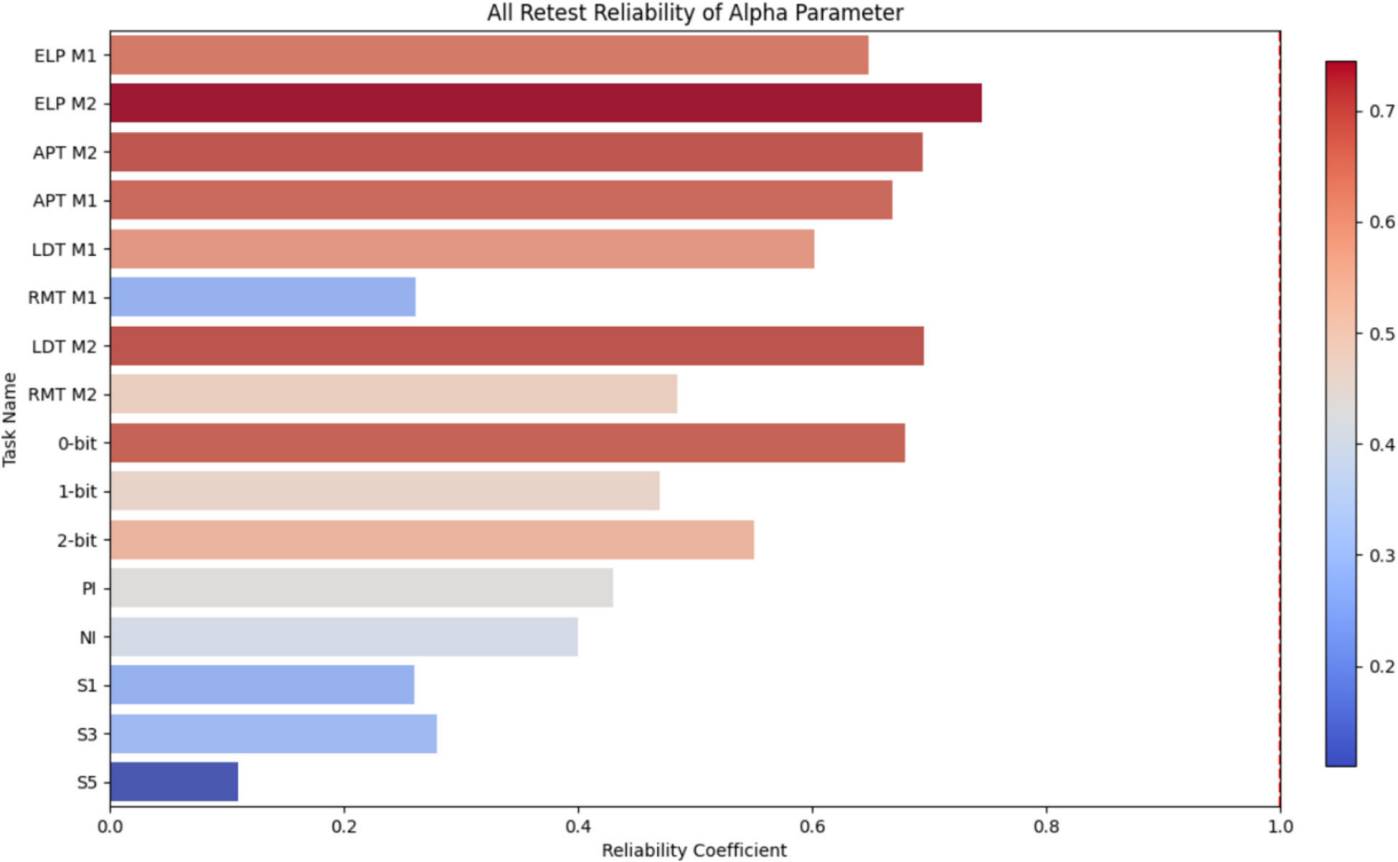
Bar graph showing the retest reliability of the alpha parameter in LFM across all the tasks (see how we coded tasks in Table 1). M1 stands for Model 1 and M2 represents Model 2

### Test–retest reliability of LFM parameters

For each correlational analysis, bivariate outliers were identified using the Mahalanobis distance (D2). Participants with extreme values ($p < 0.001$) were excluded from the respective analysis. This led to the exclusion of up to six participants across the datasets, with a maximum of 20 participants excluded from the ELP dataset, which consisted of 805 participants. Test–retest reliability coefficients (Pearson correlations) were calculated between the two sessions for the LFM parameters. In addition to the LFM parameters, reliability coefficients were also computed for accuracy rates and mean RTs for all datasets except Schubert’s study. These results are visualized in Figs. 2 and 3.

Figure 3 shows the reliability of the alpha parameter across all tasks. Except for Schubert’s S5 task, all reliability coefficients were statistically significant and, in most cases, demonstrated acceptable reliability. The highest reliability was observed in the ELP dataset across all parameters, which again supports that with the growth of the number of trails, reliability measures increase (American Educational Research Association et al., 2014). Another observable pattern is the higher reliability of the simpler model’s (Model 2) parameters. This pattern aligns with previous findings from Lerche and Voss (2017).

Similar to the study by Lerche and Voss (2017), reliability coefficients were also reported for the difference ($v_{\text {total}} = v1 - v2$) and sum ($v_{\text {bias}} = v1 + v2$) of the two drift rates. The difference can be interpreted as a measure of the overall speed of information processing, as v1 is positive and v2 is negative, reflecting the ability to distinguish between stimulus types. On the other hand, the sum represents a potential bias in drift rate or a general preference in information accumulation for one type of information. The $v_{\text {total}}$ measure showed particularly high reliability, with coefficients exceeding 0.7. While the reliability of single drift rates was somewhat lower than $v_{\text {total}}$, they remained within acceptable ranges of high values ($r > 0.6$). The threshold parameter also exhibited high reliability, though slightly lower than the drift rates.

**Table 2:** Test–retest reliability coefficients for LFM parameters across the LDT and RMT M1 and M2 refer to Model 1 and Model 2, respectively

Parameters	M1 S1	M1 S2	M2 S1	M2 S2
v1	0.232*	0.252*	0.28**	0.337***
v2	0.109	0.329**	0.065	0.25*
$v_{\text {total}}$	0.186	0.323**	0.192	0.307**
$v_{\text {bias}}$	0.118	0.12	0.23*	0.268**
zr	0.259**	0.28**	$-$	$-$
a	0.536***	0.477***	0.203*	0.299**
ndt	0.343***	0.486***	0.442***	0.427***
sndt	0.206*	0.242*	$-$	$-$
alpha_T	0.218*	0.257**	0.323**	0.483***
mean_log_rt	0.454***	0.507***	0.454***	0.507***
accuracy_T	0.338***	0.366***	0.338***	0.366***

Note that _T stands for the transformed version of the parameter distribution. Stars indicate the level of significance: * *p* value $< 0.05$, ** *p* value $< 0.01$, *** *p* value $< 0.001$

When comparing the reliability of LFM parameters to “standard” measures such as mean RT and accuracy rate, accuracy generally outperformed LFM parameters, except for the ELP dataset’s $v_{\text {total}}$. Meanwhile, mean RT performed worse than some LFM parameters, particularly $v_{\text {total}}$. The non-decision time parameter displayed lower reliability than drift rates and thresholds, although the differences were not substantial.

Additionally, across-task correlations within a single session were computed for the LFM parameters. These are essentially lower than the test–retest correlations. When it comes to alpha, ECTs did not have statistically significant across-task correlations for alpha, whereas LDT and RMT do. Detailed across-task correlation results for LDT and RMT are presented in Table 2, and for ECTs, they can be found in Table B1. The reliability of the alpha parameter across all tasks is also depicted in the bar graph in Fig. 3.

**Fig. 4:**
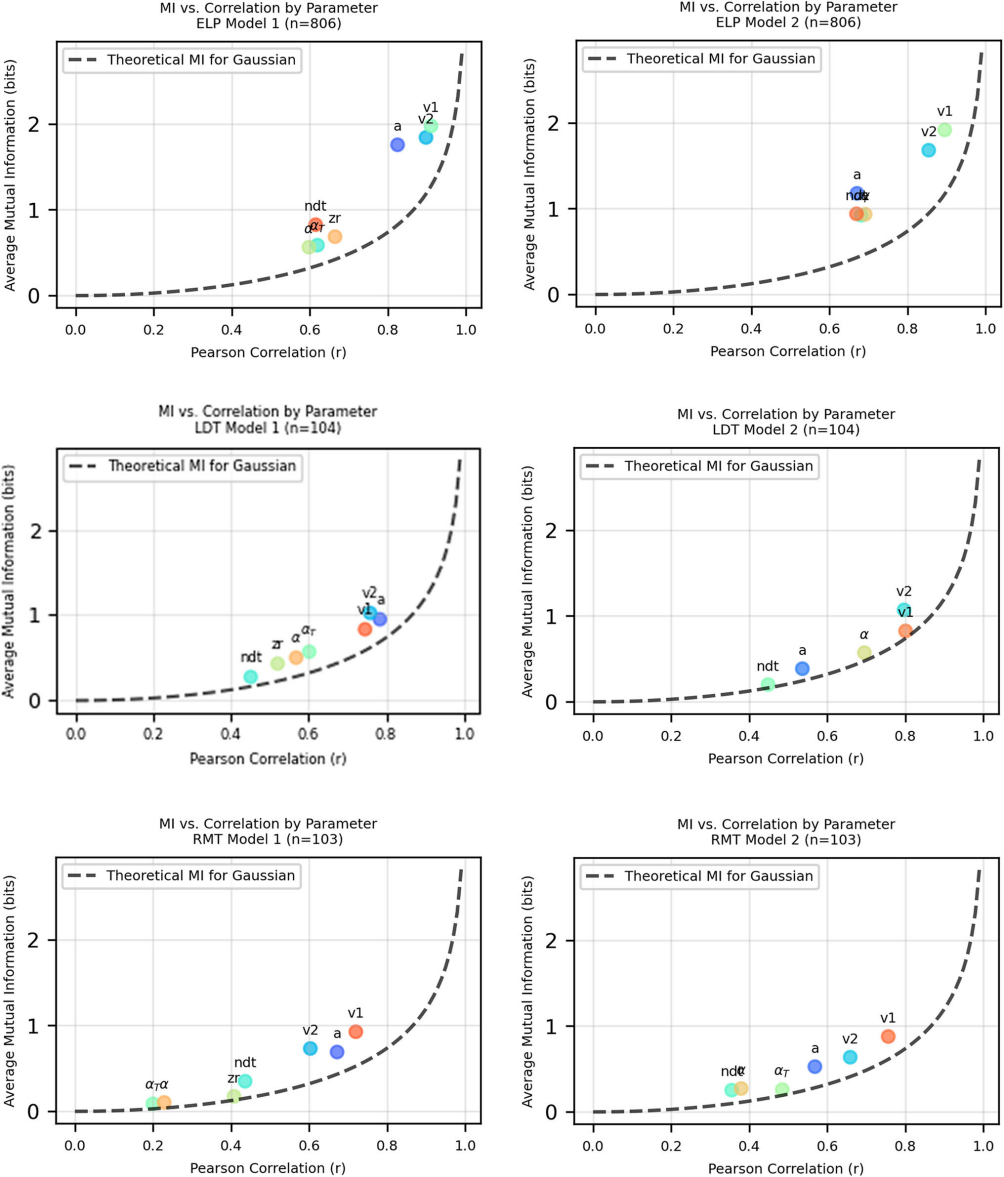
The *dashed line* shows theoretical mutual information for Gaussian-distributed variables. Most parameters are positioned above this line, indicating they contain more information than would be expected from a simple Gaussian relationship with the same correlation coefficient. The *y*-axis represents the average mutual information calculated across different values of *k*, where *k* refers to the number of nearest neighbors (k-NN) used in the analysis

**Fig. 5:**
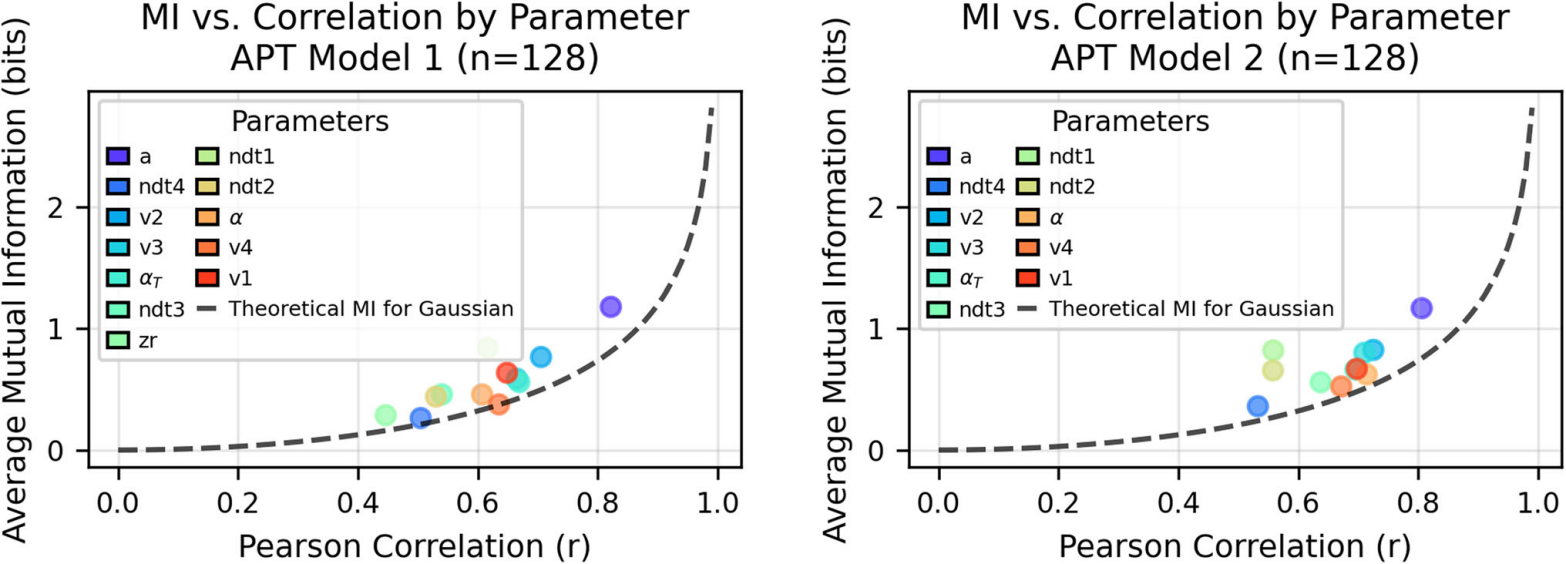
The *dashed line* shows theoretical mutual information for Gaussian-distributed variables. Most parameters are positioned above this line, indicating they contain more information than would be expected from a simple Gaussian relationship with the same correlation coefficient. The *y*-axis represents the average mutual information calculated across different values of *k*, where *k* refers to the number of nearest neighbors used in the analysis

While Pearson correlation coefficients provide a valuable measure of linear relationships between variables, they are inherently limited in their capacity to detect non-linear dependencies. MI (Cover & Thomas, 2006; Kraskov et al., 2004), rooted in information theory (Shannon & Weaver, 1949), transcends these limitations by quantifying the total amount of shared information between variables, regardless of whether the relationship is linear, non-linear, or even non-monotonic. As illustrated in Figs. 4 and 5, even parameters with comparable Pearson correlation values can exhibit markedly different mutual information quantities. The theoretical reference curve for Gaussian distributions (dashed line) demonstrates this distinction, as most parameters position themselves above this benchmark, indicating richer statistical dependencies than would be expected from simple linear relationships. Consequently, mutual information analysis reveals nuanced interdependencies between test and retest measurements that remain undetected by correlation analysis, providing a more comprehensive characterization of parameter reliability in LFM.

**Table 3:** Results of the *t* test for the transformed alpha parameter across all datasets

Task (Model)	mean_s1	std_s1	mean_s2	std_s2	*t*	dof	*p*-val	CI95%	cohen-d
LDT Model 1	0.2242	0.2151	0.1215	0.2011	5.6254	103	$<0.0001$	[0.07 0.14]	0.4933
LDT Model 2	0.0597	0.2226	-0.0196	0.209	4.7955	103	$<0.0001$	[0.05 0.11]	0.3673
RMT Model 1	0.1992	0.2209	0.1202	0.2301	3.4997	102	0.0007	[0.03 0.12]	0.3502
RMT Model 2	0.3969	0.2121	0.1665	0.1634	9.7216	102	$<0.0001$	[0.18 0.28]	1.2169
APT Model 1	0.1866	0.2274	0.2124	0.214	-1.6192	127	0.1079	[-0.06 0.01]	0.1167
APT Model 2	0.0972	0.2311	0.0793	0.2248	1.1328	127	0.2594	[-0.01 0.05]	0.0782
ELP Model 1	0.2491	0.2816	0.2067	0.2677	5.0196	805	$<0.0001$	[0.03 0.06]	0.1543
ELP Model 2	-0.1624	0.269	-0.156	0.2734	-0.8376	805	0.4025	[-0.02 0.01]	0.0236

The table displays the mean and standard deviation for session 1 (mean_s1, std_s1) and session 2 (mean_s2, std_s2), along with the *t*-value, degrees of freedom (dof), *p* value (*p*-val), 95% confidence interval (CI95%), and Cohen’s *d* effect size (cohen-d) for each task and model. Significant *p* values are indicated to highlight the presence of practice effects

### Practice effect on cognitive performance

The *t* test analysis demonstrates a significant practice effect from the first to the second session. As anticipated, our findings were similar to those reported in Lerche and Voss (2017), except for the ECTs, which are less demanding and thus were not expected to demonstrate a significant practice effect.

The impact of the practice is evident in the augmentation of information processing, reflected by an increase in drift rate and a reduction in both motor and encoding times, as indicated by decreased non-decision time. Additionally, participants adopted a more liberal decision criterion in the second session, reflected in a lower threshold. This trend was consistent across all tasks, except for the ECTs, and along the two models conveying in all tasks, indicating that participants generally performed better in the second session, aligning with previous studies (Lerche & Voss, 2017).

The practice effect on the alpha parameter is observed to be subtractive. Table 3 presents detailed results of the *t*-test analysis for the transformed alpha parameter across all datasets. This finding can also be interpreted as a “lower alpha” (more jumps) corresponds to better performance. The means and standard deviations for Session 1 and Session 2, along with *t*-values, degrees of freedom, *p* values, 95% confidence intervals, and Cohen’s d effect sizes for each task and model, are reported. In the LDT, both Model 1 and Model 2 showed significant reductions in the alpha parameter from Session 1 to Session 2, with *t*-values of 5.63 and 4.80, respectively, and *p* values less than 0.0001. The RMT also demonstrated significant practice effects in both models, with *p* values of 0.0007 for Model 1 and less than 0.0001 for Model 2. Additionally, ELP Model 1 showed a significant subtractive practice effect on the alpha parameter, with a *t*-value of 5.02 and a *p* value below 0.001. While APT Model 1, APT Model 2, and ELP Model 2 also displayed reductions in alpha across sessions, these decreases are not statistically significant, as indicated by *p* values exceeding 0.05.

Overall, the results emphasize the effect of practice in augmenting cognitive performance. For further details on the *t* test results, please refer to Appendix D, in the section titled ’*t* test Results’.

**Fig. 6:**
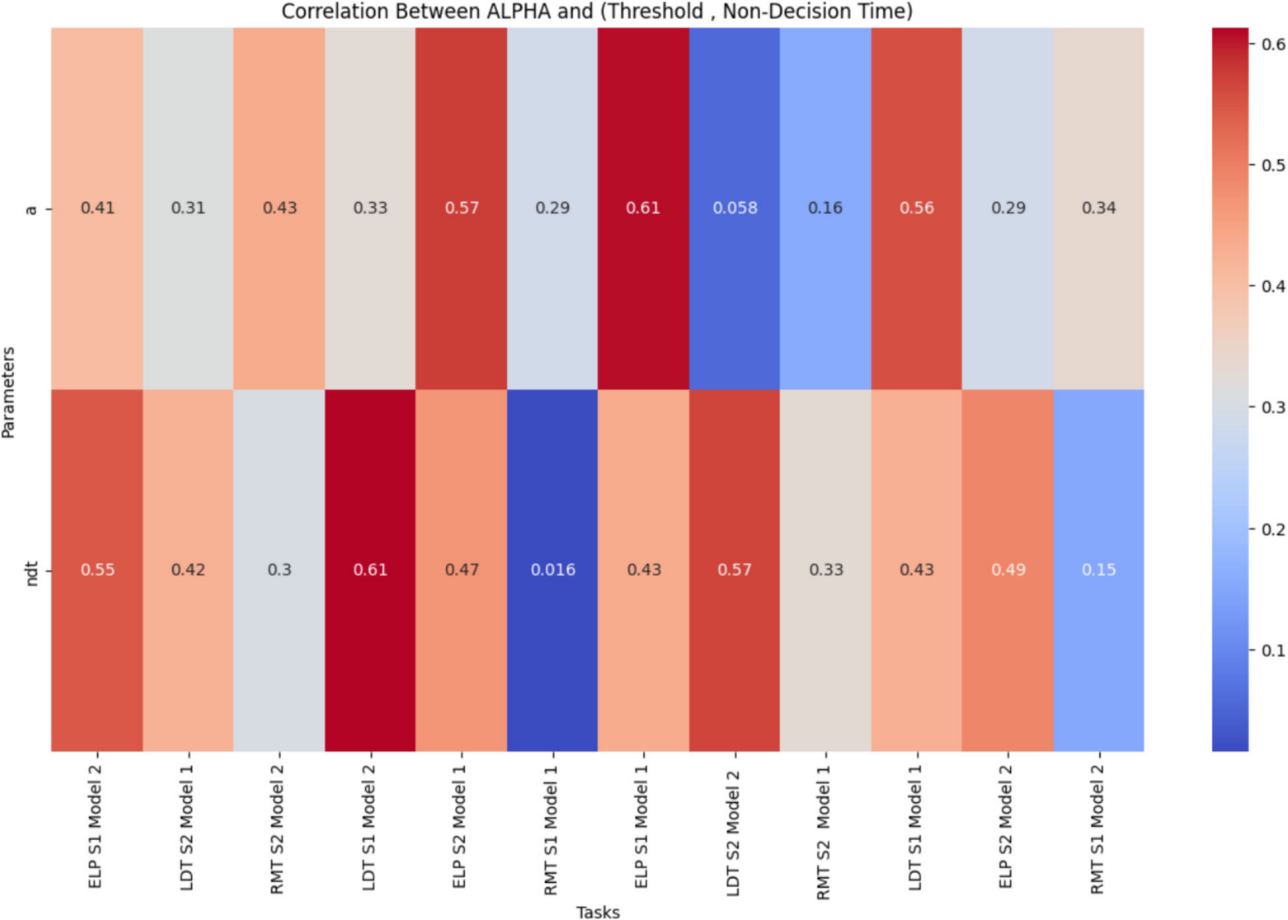
Heatmap depicting the correlation coefficients between the alpha parameter and both decision criteria (*top*) and non-decision time (*bottom*), across all tasks and models, excluding the APT task

**Fig. 7:**
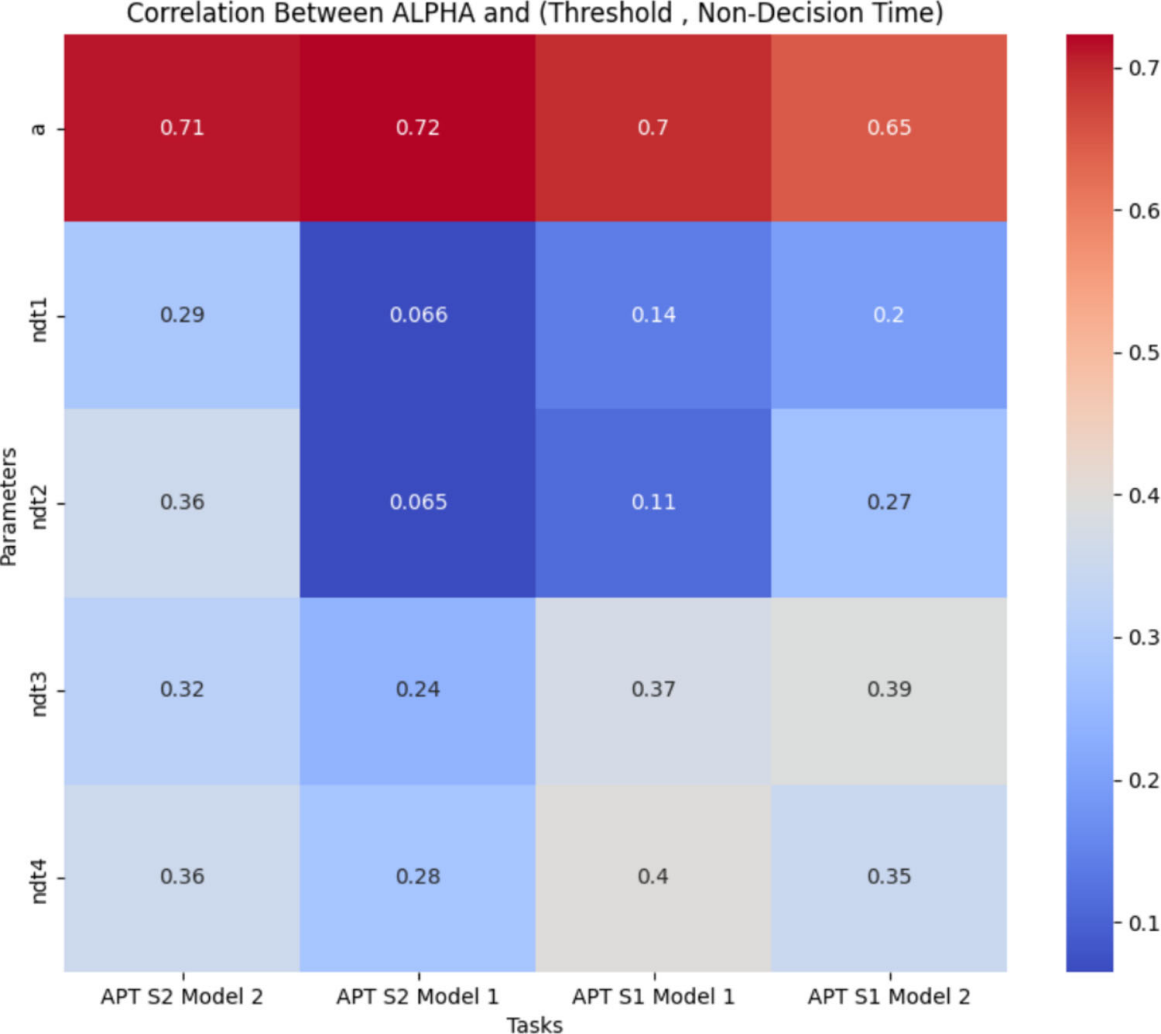
Heatmap depicting the correlation coefficients between the alpha parameter and both decision criteria (*top*) and non-decision time (*bottom*), across two models specifically for the APT Task

### Discerning whether LFM parameters represent distinct aspects of data

The correlation among the LFM parameters suggests that they largely capture distinct aspects of the data, with minimal overlap in the factors they measure or influence. The generally low and mostly insignificant correlation coefficients indicate that each parameter reflects unique facets of cognitive processes.

However, the alpha parameter, the focal point of this study, stands out as an exception. It consistently exhibits a notable correlation with both non-decision time and decision criteria across the majority of datasets (see Figs. 6 and 7). As illustrated in Fig. 6, the heatmap shows that alpha is positively correlated with non-decision time, indicating that higher alpha values are associated with longer non-decision times.

Additionally, alpha showed a significant positive correlation with decision thresholds across tasks. In Fig. 6, the heatmap highlights this relationship, revealing that lower threshold values correspond to lower alpha values.

Figure 7 further elucidates the correlations specific to the APT, demonstrating similar trends. In this figure, the correlation coefficients between alpha and both non-decision time and decision thresholds are presented, confirming the previously observed patterns.

The strong relationship between alpha and these parameters can undermine confidence regarding the reliability of alpha. Some people could argue that the alpha’s reliability is **inherited** from other reliable parameters. Furthermore, it is highly likely that the alpha does not possess any additional information beyond what has already been captured by the other parameters.

These findings underscore the importance of examining the interrelationships among LFM parameters. To gain a deeper understanding of these relationships, further regression analyses will be conducted in the following. For a detailed examination of the correlations among other parameters, readers are directed to the Appendix, specifically in the section titled ’Correlation Among LFM Parameters’ (see Appendix E)

### Investigating the intrinsic reliability of alpha

#### Gradient boosting regression (GBR)

**Fig. 8:**
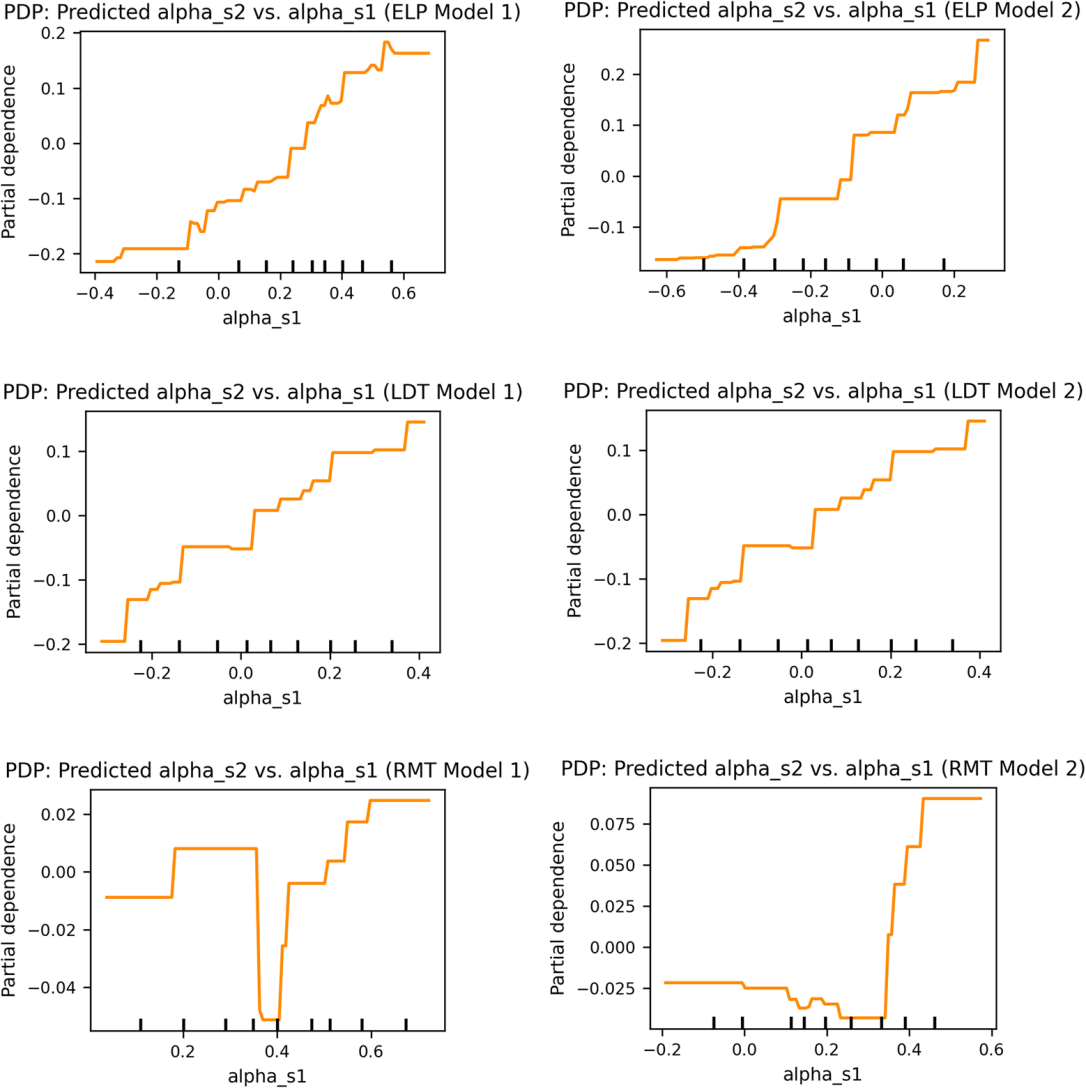
PDPs for predicting $\alpha _{s2}$ from $\alpha _{s1}$ while controlling for threshold (*a*) and non-decision time (*ndt*) parameters across tasks and models using GBR

**Fig. 9:**
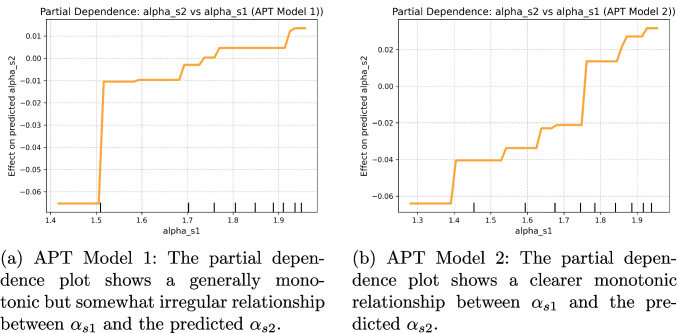
PDPs for predicting $\alpha _{s2}$ from $\alpha _{s1}$ while controlling for threshold (*a*) and non-decision time (*ndt*) parameters for APT task, across two models using GBR

We examined whether the test–retest reliability observed for the alpha parameter reflects an intrinsic source of variance or is instead inherited from its correlation with other model parameters (threshold and non-decision time). To this end, we applied a gradient boosting regression model (Friedman, 2001; Pedregosa et al., 2011) for each dataset and parameter estimation variant. The GBR model predicted alpha from session 2 ($\alpha _{s2}$) using session 1 alpha ($\alpha _{s1}$), session 1 and session 2 thresholds ($a_{s1}$, $a_{s2}$), and session 1 and session 2 non-decision times ($ndt_{s1}$, $ndt_{s2}$). In the APT dataset specifically, four separate non-decision time parameters (*ndt*1, *ndt*2, *ndt*3, *ndt*4) were explicitly included. This approach was preferred over linear regression due to the potential for non-linear relationships induced by the transformation applied to the alpha parameter. Tables 4 and 5 as well as Figs. 8 and 9 summarize the results.

**Table 4:** Gradient boosting regression results for All models

	ELP M1	ELP M2	LDT M1	LDT M2	RMT M1	RMT M2
Mean Test $R^{2}$	0.6509	0.6118	0.4388	0.5733	-0.2523	0.1166
Std Test $R^{2}$	0.0688	0.0421	0.1433	0.1523	0.2221	0.4855
Mean Test MAE	0.1140	0.1194	0.1214	0.1125	0.1869	0.1803
Mean Test RMSE	0.1564	0.1697	0.1477	0.1438	0.2297	0.2344
Imp_alpha_s1	0.5592	0.4694	0.4807	0.4679	0.0895	0.1604
ImpStd_alpha_s1	0.0270	0.0135	0.0473	0.0597	0.0165	0.0382
Imp_a_s1	0.2588	0.0400	0.0489	0.0716	0.1531	0.1201
ImpStd_a_s1	0.0096	0.0033	0.0093	0.0175	0.0474	0.0427
Imp_a_s2	0.6558	0.1894	0.1379	0.2381	0.1686	0.2561
ImpStd_a_s2	0.0433	0.0109	0.0194	0.0297	0.0635	0.0812
Imp_ndt_s1	0.1141	0.0243	0.0540	0.0359	0.0449	0.1356
ImpStd_ndt_s1	0.0069	0.0034	0.0061	0.0145	0.0320	0.0480
Imp_ndt_s2	0.3933	0.3199	0.2924	0.1863	0.4440	0.4168
ImpStd_ndt_s2	0.0181	0.0110	0.0401	0.0383	0.0981	0.1264

$R^{2}$ = Coefficient of determination, MAE = mean absolute error, RMSE = root mean squared error, Imp = permutation importance, Std = standard deviation

**Table 5:** Gradient boosting regression results explicitly including all four separate NDT parameters

	APT Model 1	APT Model 2
Mean Test $R^{2}$	0.3861	0.5099
Std Test $R^{2}$	0.2476	0.1034
Mean Test MAE	0.0757	0.1013
Std Test MAE	0.0176	0.0123
Mean Test RMSE	0.1119	0.1297
Std Test RMSE	0.0243	0.0154
Imp_alpha_s1	0.1857	0.2602
ImpStd_alpha_s1	0.0303	0.0353
Imp_a_s1	0.1857	0.2602
ImpStd_a_s1	0.0303	0.0353
Imp_a_s2	0.0536	0.0018
ImpStd_a_s2	0.0138	0.0008
Imp_ndt1_s1	0.6304	0.4370
ImpStd_ndt1_s1	0.0807	0.0450
Imp_ndt1_s2	0.0077	0.0144
ImpStd_ndt1_s2	0.0027	0.0049
Imp_ndt2_s1	0.0040	0.0249
ImpStd_ndt2_s1	0.0016	0.0050
Imp_ndt2_s2	0.0138	0.0257
ImpStd_ndt2_s2	0.0038	0.0066
Imp_ndt3_s1	0.0015	0.0181
ImpStd_ndt3_s1	0.0002	0.0063
Imp_ndt3_s2	0.0258	0.0166
ImpStd_ndt3_s2	0.0081	0.0046
Imp_ndt4_s1	0.0197	0.0011
ImpStd_ndt4_s1	0.0080	0.0004
Imp_ndt4_s2	0.0090	0.0170
ImpStd_ndt4_s2	0.0034	0.0039

$R^{2}$ = Coefficient of determination, MAE = mean absolute error, RMSE = root mean squared error, Imp = permutation importance, Std = standard deviation

For the ELP and LDT datasets, $\alpha _{s1}$ consistently emerged as one of the most important predictors of $\alpha _{s2}$, even when controlling for threshold and non-decision time. In ELP Model 1, the model achieved a mean test $R^2$ of 0.65, with $\alpha _{s1}$ ranking second in importance (0.56) after $a_{s2}$ (0.66). In ELP Model 2, $\alpha _{s1}$ was the most important predictor (0.47), with a mean test $R^2$ of 0.61. For LDT Model 1, the model explained 44% of variance ($R^2=0.44$), with $\alpha _{s1}$ again showing the highest importance (0.48). LDT Model 2 explained 57% of variance, and $\alpha _{s1}$ remained the most influential predictor (0.47). The partial dependence plots (PDPs) consistently revealed positive, monotonic relationships between $\alpha _{s1}$ and predicted $\alpha _{s2}$, reinforcing the interpretation of an intrinsic component to alpha’s reliability.

The RMT dataset showed poor fit due to the low reliability of alpha and the small trial size for this task. RMT Model 1 yielded poor prediction ($R^2=-0.25$), whereas RMT Model 2 fit was slightly better ($R^2=0.12$). The corresponding PDPs revealed weak and irregular patterns, confirming the limited predictive value of $\alpha _{s1}$ in this dataset.

In the APT dataset, both Model 1 and Model 2 showed moderate explained variance (Model 1: $R^2 = 0.39$, Model 2: $R^2 = 0.51$). The importance of $\alpha _{s1}$ was substantial (Model 1 = 0.19; Model 2 = 0.26), although it ranked below non-decision time of session 1. The PDPs for APT revealed clear monotonic relationships, further supporting the conclusion that the reliability of alpha parameter has an intrinsic component, although influenced by interactions with other parameters.

Overall, the PDP results support the idea that alpha’s reliability has an intrinsic component, even though alpha’s importance is not always the highest among predictors.

#### Conditional mutual information (CMI)

The GBR analysis reveals the contribution of each parameter in predicting $\alpha _{s2}$, but it does not quantify how much of the dependency between $\alpha _{s1}$ and $\alpha _{s2}$ is uniquely attributable to alpha, distinct from influences mediated by other parameters such as threshold or non-decision time. To address this, we employed CMI (Cover & Thomas, 2006), an information-theoretic measure that isolates the unique associations between alpha parameters across sessions.

CMI, conceptually analogous to partial dependence in regression analysis, quantifies the information shared between two variables after accounting for additional variables. It captures both linear and nonlinear dependencies in bits. To assess how much of the dependency between $\alpha _{s1}$ and $\alpha _{s2}$ is unique to alpha, we estimated both MI and CMI. The ratio of CMI to MI indicates the proportion of the dependency attributable to alpha after removing the influence of other parameters.

We estimated MI and CMI using the Kraskov estimator from the npeet Python package (Kraskov et al., 2004; Ver Steeg, 2014), calculating both unconditional MI, $I(\alpha _{s1}; \alpha _{s2})$, and conditional MI values $I(\alpha _{s1}; \alpha _{s2} \mid a_{s1}, a_{s2})$ and $I(\alpha _{s1}; \alpha _{s2} \mid ndt_{s1}, ndt_{s2})$. The ratio of conditional MI to unconditional MI provided the reliability retention index. This reliability retention index quantifies the portion of alpha’s reliability that remains after removing shared information with threshold or non-decision time parameters. A higher ratio indicates that alpha’s reliability is more intrinsic, rather than inherited from other parameters.

**Fig. 10:**
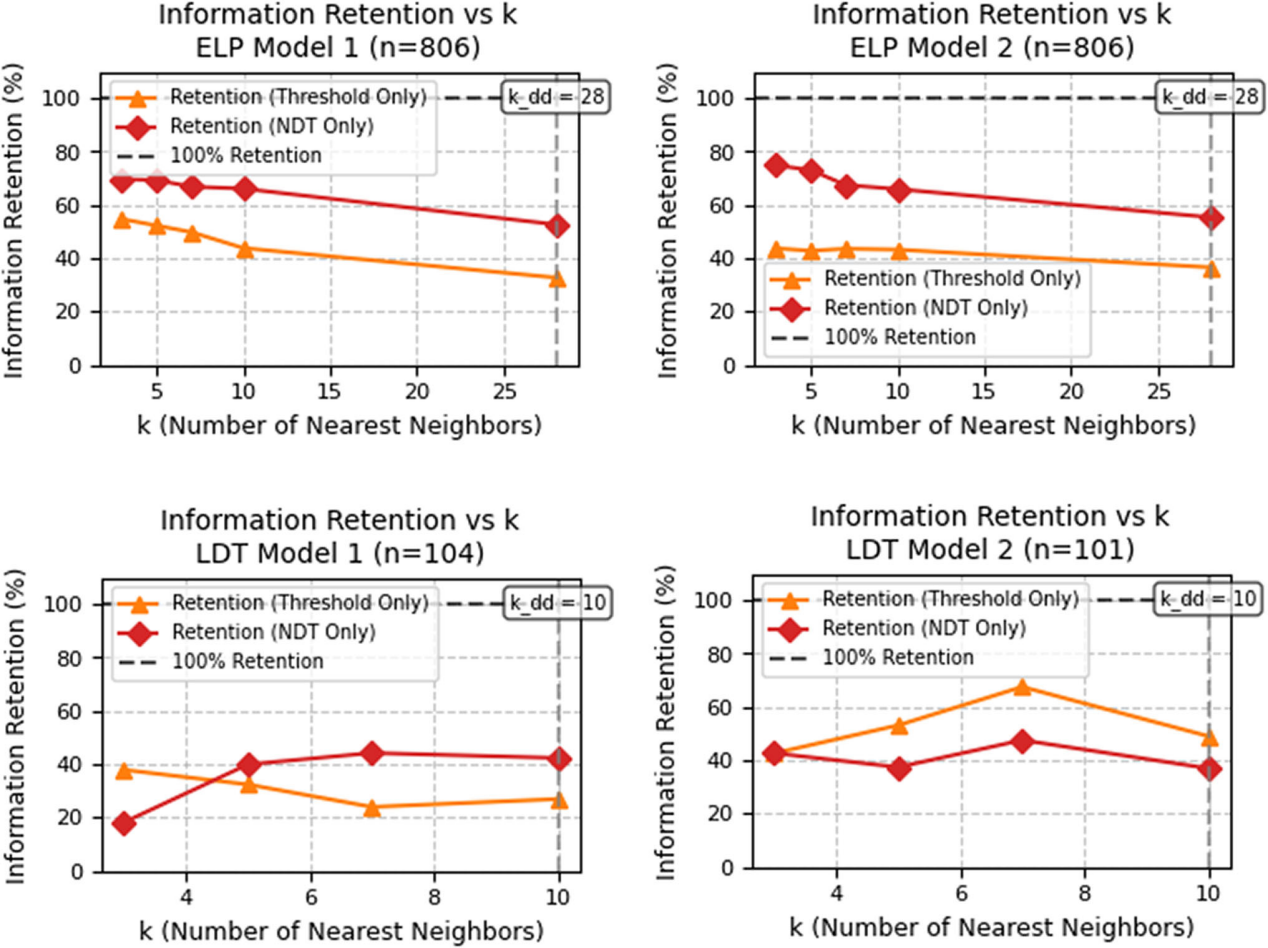
Retention of mutual information between $\alpha _{s1}$ and $\alpha _{s2}$ after conditioning on threshold (*orange*) and non-decision time (*red*) across varying *k* values. Each subplot corresponds to a specific model: ELP Model 1 and 2 (*top row*), LDT Model 1 and 2 (*bottom row*). Retention is expressed as the ratio of conditional to unconditional MI

We computed CMI for various values of *k* (number of nearest neighbors), expressing the results as a percentage of the corresponding unconditional MI. Retention values across different *k* values are shown in Fig. 10. In the ELP dataset, both models showed greater information retention after conditioning on non-decision time than on threshold. For Model 2, retention conditioned on non-decision time exceeded 55% for all *k* values, while retention conditioned on threshold dropped below 40% at higher *k* values. Model 1 showed a similar pattern, with non-decision time retaining approximately 70% of the information at low *k* values, while threshold retention started above 50% at the lowest *k* and declined at higher *k* values.

In the LDT dataset, Model 2 showed peak retention conditioned on threshold (70% at $k=7$) and lower retention conditioned on non-decision time ($\tilde{5}0$%). Model 1 had flatter retention curves across *k* values, with non-decision time surpassing threshold at higher *k*, but remaining below 50%. These results suggest that a substantial portion of the dependency remains even after conditioning, supporting the intrinsic reliability of the alpha parameter.

For the RMT dataset, CMI analysis was unreliable due to estimator limitations, consistent with previous research highlighting the sensitivity of Kraskov’s method to sample size and “weak dependencies” (Kraskov et al., 2004). In the APT dataset, we excluded conditional MI analyses due to the high-dimensional conditioning set, which led to unreliable estimations. As a result, we did not perform CMI-based retention analyses for this dataset.

### Multivariate regression analysis

#### Evaluating which aspects of data are captured by alpha

To identify which aspects of the data are reflected by the parameter alpha, we conducted a regression analysis where alpha served as the dependent variable.

Our focus centered on the transformed values of CRT, ERT, and Accuracy (Acc), with the decision threshold (*a*) and non-decision time (*ndt*) included as control variables (see Table 6).

The analysis revealed that ERT is a consistently significant predictor of $\alpha$ across all tasks, demonstrated by uniformly low *p* values and positive coefficients. For instance, in the RMT Model 2 Session 1, the ERT coefficient was 0.6135 ($p < 0.0001$), indicating a strong positive relationship. Similarly, in RMT Model 2 Session 2, the coefficient was 0.4947 ($p < 0.0001$). These results suggest that higher ERT values are associated with increased alpha.

Accuracy (Acc) also emerged as a significant predictor of alpha in several tasks. For instance, in the RMT Model 2 Session 1, the Acc coefficient was 0.4769 ($p = 0.019$), demonstrating its predictive strength. Similarly, in the LDT Model 1 Session 1, the coefficient was 0.1632 ($p = 0.03$), while in Session 2, it was 0.3767 ($p < 0.0001$). However, exceptions were noted in the RMT Model 1 Session 2 and RMT Model 2 Session 2, where the relationships did not reach statistical significance, with coefficients of 0.0515 ($p = 0.61$) and 0.186 ($p = 0.195$), respectively. This variation underscores the complexity of the relationship between accuracy and the alpha parameter, indicating that while accuracy can inform the reliability of decision-making, its influence may be context-dependent.

Additionally, CRT was found to significantly predict alpha in some cases, though the direction of its coefficients fluctuated between positive and negative. In the ELP Model 1 Session 1, the coefficient was -0.2034 ($p < 0.0001$) with an R-squared value of 0.464, indicating a negative relationship. In ELP Model 1 Session 2, CRT had a coefficient of -0.3012 ($p < 0.0001$) with an R-squared value of 0.413, reinforcing its predictive power. In the LDT Model 2, significant predictions were observed with coefficients of 0.3482 ($p = 0.005$) for Session 1 and 0.381 ($p = 0.003$) for Session 2. However, for the remaining tasks, CRT did not achieve the threshold for statistical significance ($p > 0.05$), suggesting limitations in its predictive power in certain contexts.

The threshold parameter (*a*) also showed variability in both its significance and the direction of its coefficients across tasks. For instance, in RMT Model 2 Session 1, the coefficient was -0.1703 ($p = 0.184$) and in Model 2 Session 2, it was 0.0642 ($p = 0.484$). In the LDT Model 2 Session 1, the coefficient was -0.1169 ($p = 0.146$) and in Model 2 Session 2, it was -0.2423 ($p < 0.0001$), reflecting the different decision strategies employed by participants. On the other hand, non-decision time was found to be a significant predictor of alpha specifically in the ELP. For example, in Model 1 Session 1, *ndt* had a coefficient of 0.7434 ($p < 0.0001$), and in Model 1 Session 2, it was 0.9262 ($p < 0.0001$).

Overall, these analyses provide strong evidence that alpha consistently captures error responses and accuracy across various cognitive tasks, further substantiating the inherent quality of the reliability coefficients measured in the reliability section.

#### Alpha’s influence on the shape of distribution

Hitherto, our analyses have been inspired by Voss et al. (2019), and now we are directly referring to their simulation study of the LFM. This simulation generated $10^6$ paths per each alpha value (1.0 to 2.0) to assess prediction characteristics.

Key findings from their simulation indicate that error responses tend to be faster than correct decisions, especially at lower alpha values, where errors result from large, immediate jumps in the decision process. This effect diminishes as alpha increases, aligning more closely with Gaussian noise, where the decision speeds between errors and correct responses tend to converge. A distinctive feature of the predicted decision-time distributions is their very steep lower edges, reflecting rapid decisions occurring within the first few milliseconds of the decision process. These findings, detailed in Table 7 and Figure 2 from Voss et al. (2019), also highlight that lower alpha values are associated with more variable (StD) and skewed response times.

For example, when alpha is equal to 1.0, the percent correct (pc) was 0.78, with mean decision time (DT) for correct responses at 753 ms (SD = 537, skew = 1.60) and mean DT for errors at 556 ms (SD = 519, skew = 1.90). At alpha 1.2, pc increased to 0.79, with mean DT for correct responses at 721 ms (SD = 536, skew = 1.72) and mean DT for errors at 569 ms (SD = 530, skew = 1.92). When alpha was 1.4, pc returned to 0.78, with a mean DT of 673 ms (SD = 517, skew = 1.81) for correct responses and 571 ms (SD = 516, skew = 1.87) for errors. At alpha 1.6, pc remained at 0.78, but the mean DT for correct responses dropped to 617 ms (SD = 483, skew = 1.86), and for errors, it was 561 ms (SD = 488, skew = 1.91). For alpha 1.8, pc decreased to 0.76, with mean DT for correct responses at 553 ms (SD = 439, skew = 1.92) and for errors, 532 ms (SD = 442, skew = 1.88). Finally, at alpha 2.0, pc dropped further to 0.74, with the mean DT for both correct and error responses converging at 486 ms (SD = 389, skew = 1.96 for correct responses and 1.95 for errors).

To investigate these characteristics, we conducted a regression analysis to examine the effects on the experimental data. Table 7 demonstrates that the first quartile (Q1) of CRT is a predictor of the alpha parameter. All of the Q1 coefficients are positive, except for RMT_M1_S1, where the coefficient is negative but not statistically significant ($p > 0.005$). Meanwhile, most coefficients show significant *p* values, indicating that longer reaction times in the first quartile are associated with higher alpha values.

Although the R-squared values might appear modest, an R-squared around 20% is notable as it indicates that a substantial proportion of the variability in the alpha parameter is explained by the quartiles of reaction times. This is particularly remarkable considering the inherent complexity of cognitive processes and the LFM. The insignificance of the other quartiles (Q2, Q3, Q4) substantiates that for longer response times, the constant drift eventually brings the process to the correct threshold.

The pattern for ERT detailed in Table 8 mirrors the findings for CRT. None of the Q1 coefficients for ERT is negative, and most show significant *p* values. Table 9 presents the regression model where alpha is the dependent variable and the difference between the logarithmized mean reaction times of correct responses and error responses serves as the independent variable. Despite low *p* values and negative coefficients, the minor R-squared value provides insufficient evidence to draw definitive conclusions. Likewise, we did not observe any patterns that would confirm the presence of more varied and skewed response times at lower alphas in our analysis (see Table 10). To further investigate these characteristics, it would be more informative to examine them by comparing different “groups”.

## Discussion

This study examines the reliability of the Lévy flight model parameters. Unlike the diffusion decision model, the LFM lacks a firm theoretical framework. This limitation means that its parameters, including alpha, lack intrinsic theoretical interpretations without a supporting theoretical framework.

Specifically, the LFM challenges the DDM’s assumption of finite variance in what we call Latent Perturbations, an assumption that the DDM adopts without empirical validation by modeling LPs with a normal distribution. In contrast, the LFM utilizes a stable distribution, permitting infrequent but substantial deviations (’jumps’) from the drift rate during the decision-making process (Voss et al., 2019). The probability of such extreme events is governed by an additional parameter, alpha, which characterizes the heaviness of the tails in the noise distribution. When treated as a free parameter, alpha enables the model to capture individual differences in sensitivity to LPs, thus potentially encoding meaningful psychological information.

Our investigation represents a first step in assessing the reliability of the LFM parameters. Establishing alpha’s stability across sessions and tasks could position it as an important marker, bridging computational modeling with cognitive theory.

**Table 6:** Regression analysis examining which aspects of data are captured by the alpha parameter ($\alpha$)

Data name	R-square	CRT Coef.	CRT P	ERT Coef.	ERT P	Acc Coef.	Acc P	a Coef.	a P	ndt Coef.	ndt P
RMT_M2_S1	0.317	0.1754	0.408	0.6135	$<0.0001$	0.4769	0.019	-0.1703	0.184	-0.6735	0.261
RMT_M2_S2	0.502	0.1841	0.292	0.4947	$<0.0001$	0.186	0.195	0.0642	0.484	0.6684	0.154
LDT_M2_S2	0.681	0.381	0.003	0.5336	$<0.0001$	0.532	$<0.0001$	-0.2423	$<0.0001$	-0.5555	0.158
LDT_M2_S1	0.678	0.3482	0.005	0.3259	0.002	0.3214	$<0.0001$	-0.1169	0.146	0.7532	0.089
RMT_M1_S1	0.229	-0.0551	0.794	0.4494	0.004	-0.475	0.022	0.1042	0.506	-0.9446	0.092
RMT_M1_S2	0.411	0.1773	0.206	0.3162	$<0.0001$	0.0515	0.61	-0.0833	0.344	0.7579	0.055
LDT_M1_S2	0.535	0.0664	0.663	0.7588	$<0.0001$	0.3767	$<0.0001$	-0.3288	$<0.0001$	-0.1171	0.777
LDT_M1_S1	0.53	0.1606	0.291	0.4491	0.001	0.1632	0.03	-0.0125	0.926	0.9641	0.075
ELP_M1_S1	0.464	-0.2034	$<0.0001$	0.287	$<0.0001$	0.5314	$<0.0001$	0.1794	$<0.0001$	0.7434	$<0.0001$
ELP_M1_S2	0.413	-0.3012	$<0.0001$	0.201	$<0.0001$	0.3526	$<0.0001$	0.2225	$<0.0001$	0.9262	$<0.0001$
ELP_M2_S1	0.525	0.0634	0.27	0.3461	$<0.0001$	0.7733	$<0.0001$	0.0396	0.111	0.5814	$<0.0001$
ELP_M2_S2	0.38	-0.027	0.683	0.2603	$<0.0001$	0.5879	$<0.0001$	0.0372	0.217	0.6444	$<0.0001$

The dependent variable is $\alpha$ for each task, while the independent variables include mean logarithmized CRT, mean logarithmized ERT, transformed accuracy (ACC), decision threshold (*a*), and non-decision time (*ndt*). The table presents R-squared values (R-square), coefficients (Coef.), and *p* values (P) for each predictor across all tasks. The findings indicate that $\alpha$ consistently reflects error responses and accuracy, reinforcing its reliability across multiple cognitive tasks

**Table 7:** Regression analysis results for the first quartile (Q1) of mean logarithmized CRT as a predictor of the alpha parameter ($\alpha$)

Data name	R-square	const Coef.	const *p*	Q1 Coef.	Q1 *p*	Q2 Coef.	Q2 *p*	Q3 Coef.	Q3 *p*	Q4 Coef.	Q4 *p*
RMT_M2_S1	0.106	0.2166	0.022	0.7166	0.073	-0.7982	0.157	-0.1001	0.827	0.4427	0.012
RMT_M2_S2	0.233	0.3195	0.006	0.5747	0.153	-0.9895	0.108	1.126	0.013	0.0358	0.834
LDT_M2_S2	0.233	0.4118	$<0.0001$	0.9911	0.007	-0.3314	0.505	-0.0708	0.85	0.1867	0.163
LDT_M2_S1	0.34	0.4095	$<0.0001$	0.5711	0.039	-0.1662	0.639	0.3718	0.21	0.0867	0.483
RMT_M1_S1	0.061	0.3238	0.001	-0.034	0.93	-0.2233	0.686	0.0969	0.83	0.2816	0.103
RMT_M1_S2	0.197	0.3203	$<0.0001$	0.3639	0.213	-0.3496	0.433	0.4381	0.177	0.0886	0.475
LDT_M1_S2	0.158	0.5089	$<0.0001$	1.084	0.003	-0.7641	0.129	0.2695	0.476	0.0199	0.882
LDT_M1_S1	0.312	0.5774	$<0.0001$	0.5412	0.048	0.1345	0.701	0.0185	0.949	0.1398	0.252
APT_M1_S1	0.235	0.5631	$<0.0001$	1.2856	$<0.0001$	-0.7536	0.06	0.2363	0.391	0.0993	0.436
APT_M1_S2	0.219	0.5532	$<0.0001$	1.1799	0.002	-0.7287	0.165	-0.0262	0.939	0.3274	0.012
APT_M2_S1	0.22	0.4233	$<0.0001$	1.1724	$<0.0001$	-0.7362	0.073	0.2341	0.408	0.1695	0.196
APT_M2_S2	0.265	0.4922	$<0.0001$	1.4766	$<0.0001$	-0.7198	0.178	-0.3141	0.372	0.3909	0.003
ELP_M1_S1	0.232	0.0299	0.344	0.5779	$<0.0001$	-0.1159	0.33	-0.3	0.007	0.4326	$<0.0001$
ELP_M1_S2	0.138	-0.0176	0.577	0.3504	$<0.0001$	0.0816	0.54	-0.3759	0.002	0.3533	$<0.0001$
ELP_M2_S1	0.219	0.4968	$<0.0001$	0.6385	$<0.0001$	-0.0652	0.542	-0.1656	0.097	0.1224	0.06
ELP_M2_S2	0.208	0.4628	$<0.0001$	0.5225	$<0.0001$	0.1171	0.295	-0.2017	0.051	0.0182	0.757

The table includes R-squared values, constant coefficients, and *p* (*p* values) for each quartile (Q1 to Q4) across all tasks and models. Significant positive coefficients indicate that longer reaction times in the first quartile are associated with higher alpha values

**Table 8:** Regression analysis results for the first quartile (Q1) of mean logarithmized ERT as a predictor of the alpha parameter ($\alpha$)

Data name	R-square	const Coef.	const *p*	Q1 Coef.	Q1 *p*	Q2 Coef.	Q2 *p*	Q3 Coef.	Q3 *p*	Q4 Coef.	Q4 *p*
RMT_M2_S1	0.286	0.2123	0.024	0.5201	0.129	-0.3959	0.298	0.3141	0.209	0.1827	0.197
RMT_M2_S2	0.611	0.0727	0.309	0.0306	0.881	0.296	0.298	0.0901	0.691	0.42	0.003
LDT_M2_S2	0.472	0.2372	0.026	0.8896	0.001	-0.9477	0.012	0.3701	0.156	0.2854	0.014
LDT_M2_S1	0.496	0.3067	0.004	0.5618	0.043	0.0349	0.923	-0.3128	0.206	0.4784	$<0.0001$
RMT_M1_S1	0.172	0.5726	$<0.0001$	0.7465	0.033	-0.2271	0.555	-0.0091	0.971	0.0438	0.76
RMT_M1_S2	0.465	0.4043	$<0.0001$	0.4899	0.007	0.1145	0.638	0.0177	0.927	-0.0014	0.99
LDT_M1_S2	0.512	0.652	$<0.0001$	1.3693	$<0.0001$	-1.1242	0.002	0.482	0.049	0.0454	0.67
LDT_M1_S1	0.492	0.5496	$<0.0001$	0.6035	0.024	0.0334	0.924	-0.163	0.492	0.3199	0.005
APT_M1_S1	0.437	0.6498	$<0.0001$	1.1639	$<0.0001$	-0.3623	0.4	0.0719	0.808	0.2485	0.065
APT_M1_S2	0.423	0.6784	$<0.0001$	0.901	0.001	0.0451	0.924	-0.2474	0.531	0.3534	0.007
APT_M2_S1	0.321	0.4341	$<0.0001$	0.9656	0.001	-0.4448	0.358	0.1045	0.753	0.3086	0.041
APT_M2_S2	0.324	0.5329	$<0.0001$	0.7573	0.013	0.2886	0.596	-0.3554	0.433	0.3184	0.033
ELP_M1_S1	0.334	0.4965	$<0.0001$	0.5863	$<0.0001$	-0.1143	0.201	0.1174	0.171	-0.039	0.506
ELP_M1_S2	0.306	0.434	$<0.0001$	0.6471	$<0.0001$	-0.3267	0.002	0.1327	0.18	0.0445	0.447
ELP_M2_S1	0.311	-0.0353	0.195	0.3738	$<0.0001$	0.0371	0.716	-0.1872	0.056	0.3758	$<0.0001$
ELP_M2_S2	0.234	-0.0956	0.001	0.4469	$<0.0001$	-0.4356	$<0.0001$	-0.0082	0.945	0.452	$<0.0001$

The table includes R-squared values, constant coefficients, and *p* (*p* values) for each quartile (Q1 to Q4) across all tasks and models

**Table 9:** Regression analysis results showing the relationship between the alpha parameter ($\alpha$) and the difference between correct and error reaction times (RT.diff)

Data name	R-squared	const Coef.	const *p* value	RT_diff Coef.	RT_diff *p* value
RMT_M2_S1	0.146	-0.4411	0.006	-0.5133	$<0.0001$
RMT_M2_S2	0.17	-0.7343	$<0.0001$	-0.7181	$<0.0001$
LDT_M2_S2	0.083	-0.6204	0.003	-0.5441	0.004
LDT_M2_S1	0.033	-0.304	0.119	-0.329	0.07
RMT_M1_S1	0.085	-0.0784	0.623	-0.3817	0.004
RMT_M1_S2	0.075	-0.2399	0.113	-0.3442	0.008
LDT_M1_S2	0.145	-0.6344	0.001	-0.6846	$<0.0001$
LDT_M1_S1	0.053	-0.2156	0.248	-0.4013	0.022
APT_M1_S1	0.008	-0.0029	0.988	-0.1923	0.315
APT_M1_S2	0.003	0.0649	0.801	-0.1368	0.575
APT_M2_S1	$<0.0001$	0.0579	0.767	-0.0412	0.833
APT_M2_S2	$<0.0001$	0.0708	0.793	-0.0045	0.986
ELP_M1_S1	0.023	-0.1446	0.066	-0.2949	$<0.0001$
ELP_M1_S2	0.03	-0.2423	0.003	-0.3462	$<0.0001$
ELP_M2_S1	0.011	-0.4459	$<0.0001$	-0.2261	0.003
ELP_M2_S2	0.024	-0.5772	$<0.0001$	-0.3529	$<0.0001$

The table includes R-squared values, constant coefficients (const Coef.), coefficients (Coef.), and *p* values for each task and model

Utilizing data from three previous studies (including 12 tasks) (Yap et al., 2012; Lerche & Voss, 2017; Schubert et al., 2016), we evaluated the reliability of the LFM parameters with a main focus on the stability parameter alpha, under the “assumption”^3^ that such a theoretical framework that accounts for jumps in the process exists. Our objective was to provide evidence supporting the existence of such a framework and the significance of the alpha parameter. Our comprehensive analysis comprised the following key steps: First, we conducted a reliability and generalizability analysis to test the stability of these parameters across sessions and tasks. We also examined the impact of practice on information processing and performance. Next, we engaged in an analysis named “Discern if Parameters Represent Different Aspects of Data”, examining if each parameter uniquely reflects distinct aspects of the data. This was followed by an investigation into the “Intrinsic Reliability of Alpha” through GBR and CMI analyses. We then explored “Which Aspect of Data is being Captured by Alpha”, using regression analyses, and concluded with an analysis of the features of the predicted decision-time distribution to understand the dynamics of rapid decision-making. These steps allowed us to investigate the intrinsic reliability and predictive capacity of alpha, reflecting its critical role in understanding cognitive processes within decision-making tasks.

### Reliability and generalizability analysis

Our analysis began with the evaluation of test–retest reliability coefficients for all parameters, including alpha, across all 12 tasks and two models. The results were statistically significant for all but alpha in one task (i.e., S5^4^). Specifically, the reliability hierarchy was most notable for drift, followed by the threshold, non-decision time, and finally alpha. This order underscores the stability of these parameters across two sessions. Across-tasks correlations within a single session were also calculated, revealing a similar level of generalizability for the LFM parameters as observed for the DDM parameters in the original studies, which we reanalyzed. Note that the original studies using the DDM did not include the stability parameter alpha, but the generalizability of the other corresponding parameters is what we are referring to in this comparison.

For alpha, across-task reliability coefficients were significant in demanding tasks such as LDT and RMT within the same session but not in simpler tasks such as ECTs. This suggests that alpha may also capture elements such as strategy use and cognitive adaptations that are more prevalent in challenging tasks.

**Table 10:** Regression analysis results assessing the relationship between the alpha parameter ($\alpha$) and the skewness and standard deviation (StD) of CRT

Data name	R-square	const Coef.	const p	StD Coef.	StD p	skew Coef.	skew p
RMT_M2_S1	0.071	0.0942	0.033	0.3346	0.086	0.0348	0.265
RMT_M2_S2	0.125	0.0465	0.27	0.8306	$<0.0001$	-0.0818	0.008
LDT_M2_S2	0.039	-0.0757	0.049	0.3421	0.075	-0.0073	0.79
LDT_M2_S1	0.159	-0.0432	0.329	0.6461	$<0.0001$	-0.0493	0.059
RMT_M1_S1	0.071	0.3151	$<0.0001$	0.498	0.009	-0.0191	0.522
RMT_M1_S2	0.093	0.1149	$<0.0001$	0.5158	0.002	-0.045	0.044
LDT_M1_S2	0.008	0.0946	0.013	0.1154	0.537	0.0041	0.879
LDT_M1_S1	0.128	0.1195	0.007	0.5543	$<0.0001$	-0.0301	0.24
APT_M1_S1	0.014	0.1373	0.002	0.1778	0.346	0.0076	0.789
APT_M1_S2	0.068	0.1234	0.001	0.3909	0.091	0.0194	0.434
APT_M2_S1	0.027	0.0258	0.551	0.2638	0.167	0.0093	0.744
APT_M2_S2	0.086	-0.0194	0.598	0.3188	0.184	0.039	0.132
ELP_M1_S1	0.012	0.148	$<0.0001$	0.1453	0.028	0.0196	0.104
ELP_M1_S2	0.006	0.1232	$<0.0001$	0.1377	0.032	0.002	0.854
ELP_M2_S1	0.075	-0.3195	$<0.0001$	0.4707	$<0.0001$	0.0419	0.001
ELP_M2_S2	0.05	-0.2769	$<0.0001$	0.4312	$<0.0001$	0.0186	0.127

The table presents R-squared values, constant coefficients, and *p* values for each predictor across all tasks and models

### Practice effect

Voss et al., inspired by the notion that two-dimensional Lévy flights can enhance animal foraging efficiency, propose that these patterns could similarly reflect effective decision-making processes in “**certain**” situations. They argue that frequent shifts between different perceptual hypotheses could lead to substantial fluctuations in decision-making, indicative of a dynamic and efficient style. Supporting this idea, preliminary and unpublished findings from their lab suggest a correlation where lower stability in decision processes is associated with higher intelligence and impulsivity, alongside increased negative emotionality in borderline patients. Extending this argument, a highly stable diffusion process may indicate inefficient decision-making, characterized by a decision-maker’s inability to swiftly alternate attention among various potential targets.

The results of the *t*-test analysis from the current study support their hypothesis. To determine the practice effects from session 1 to session 2, our *t*-test analysis demonstrated that the impact of practice becomes evident through the augmentation of information processing, represented by the increase in drift rate, and the reduction of motor time and encoding, conveyed by the non-decision time. As a result of the decreased threshold, participants adopted a more liberal criterion in the second session. This pattern is consistent across all tasks and models, with participants performing better in the second session for all tasks except for ECTs, which we did not expect. The practice effect on alpha was subtractive, indicating that a “lower alpha” (more jumps) corresponds to better performance.

Wieschen et al. (2023) found that older adults demonstrate more gradual evidence accumulation, reflected by higher alpha values (or less ’jumpy’ decision processes), aligning with our findings that lower alpha values correspond to better performance. While it is well known that older adults generally perform worse due to a more conservative response style, reflected by a higher decision threshold in the DDM, our findings suggest that alpha, in the LFM, is also an important factor in understanding performance, with lower alpha values indicating more efficient decision-making.

Rasanan et al. (2021) explored the practice effect on the alpha parameter within a single session. Their findings revealed no significant differences in alpha between blocks, though they observed a gradual upward trend.

### Discerning if parameters represent different aspects of data

In progressing from a broad examination of reliability to a more targeted analysis, we excluded ECTs to focus on six tasks across two models that are more demanding. By analyzing the correlation among the parameters to determine whether they represent different aspects of the data, we also rigorously questioned our observation of their reliability. The generally low and often insignificant correlations suggested that each parameter uniquely represents different aspects of the data. However, the alpha parameter was an exception; it showed significant correlations with both non-decision time and threshold, which were sometimes considerable. This raised concerns about the observed reliability of the alpha parameter, as it may have inherited its stability from these other reliable parameters.

### Intrinsic reliability of alpha and its relation with non-decision time and threshold

The GBR analyses provided robust evidence that the observed test–retest reliability of the alpha parameter includes an intrinsic component, beyond correlations with threshold and non-decision time parameters. In both the ELP and LDT datasets, alpha from session 1 ($\alpha _{s1}$) consistently emerged among the most influential predictors for session 2 alpha ($\alpha _{s2}$), explaining substantial variance (e.g., mean $R^2$ ranging from 0.44 to 0.65 across models). PDPs further supported this conclusion, illustrating clear positive monotonic relationships between $\alpha _{s1}$ and predicted $\alpha _{s2}$.

Complementing these GBR findings, CMI analyses quantitatively isolated the unique variance in the alpha parameters across sessions. The ratio of CMI to unconditional MI provided a reliability retention index, which quantifies how much of alpha’s reliability is not shared with other parameters, such as threshold or non-decision time, and is unique to alpha. In the ELP dataset and LDT Model 1, conditioning on non-decision time resulted in higher retention compared to threshold. In contrast, the LDT dataset with Model 2 showed peak retention for threshold (approximately 70% at $k = 7$) and lower retention for non-decision time ($\tilde{5}0$%). In the RMT dataset, CMI was unreliable due to limitations in the estimator, and in the APT dataset, high-dimensional conditioning led to unreliable estimates. Therefore, CMI analyses were excluded for these datasets.

Together, the GBR and CMI analyses supported that a substantial portion of the reliability of alpha is intrinsic. Correlation among parameters of a model also points to shared underlying mechanisms. Consequently, these results also suggested that “common” cognitive factors may influence both the non-decision and decision phases, affecting both the initial encoding and the noise characteristics in the decision-making processes.

### Which aspect of data is being captured by alpha

Another regression model, aimed at investigating which aspect of the data is being captured by alpha, revealed that alpha has a consistent strong positive relation with the mean reaction time of the error responses rather than the correct responses.

In this model, alpha was dependent and the mean reaction time of the correct and error responses along with accuracy, threshold, and non-decision time were independent variables. The coefficients of the accuracy were also, in most cases, significant and positive, suggesting inaccurate performance of participants with lower alpha. The absence of a permanent pattern pertaining to the threshold and non-decision time in this model prevents any definitive conclusion, but when the coefficients are significant they are also positive. The key finding of this analysis was the consistently positive and highly significant coefficients of the error reaction times. This observation is indicative of how alpha impacts the decision-making process in the LFM, supporting findings by Voss et al. (2019) that errors typically result from large, incorrect directional jumps at the onset of decision processes. For correct responses, the consistent drift ensures that decisions reach the correct threshold, thereby prolonging mean reaction times for correct outcomes.

This characteristic of Lévy-flight models provides an account for fast error responses (Voss et al., 2019) (we will discuss this more). This finding motivated us also to examine the other features of the predicted decision-time distribution mentioned by Voss et al. (2019) in the following.

### Features of the predicted decision-time distribution

Further regression analysis confirms the presence of notably steep lower edges in the predicted decision-time distribution, indicative of rapid decision-making that occurs within the initial milliseconds of the process (Voss et al., 2019). Alpha’s predictability from the first quartile (Q1) (not from Q2, Q3) of both correct and reaction time with positive and significant coefficients in most tasks and both models approve it. Lower alpha values correspond to shorter response times in the initial quartile. Investigating other features of the predicted decision-time distribution mentioned by Voss et al., we noted that despite the presence of significant negative coefficients illustrating a reverse relationship between alpha and the difference between correct and error response times, the low R-squared values suggest that these findings do not provide a robust basis for definitive conclusions. Likewise, our analysis did not reveal any discernible patterns that would suggest more varied and skewed response times at lower alpha values.

These results suggest that the LFM parameters measure stable inter-individual differences in decision-making and potentially possess psychological significance, including parameter alpha. Alpha controls the tail of the noise distribution. By applying the LFM with alpha as a free parameter and confirming its reliability, we implicitly suggest that the unobservable internal and external factors influencing decision LPs, which are encapsulated within the model’s noise, possess distinct characteristics. This approach implies that the impact of these factors on individual decision-making processes is unique to each person, and is captured through the noise distribution encoded by the alpha parameter. As alpha decreases, deviations from the drift increase, which, unlike in DDM, allows for infrequent but large deviations from the drift. In closing, our findings not only confirmed the reliability of each parameter but also emphasized alpha’s pivotal role in characterizing response time distributions and decision-making variability under the LFM, supported by additional regression models.

### Fast errors, noise or bias

Previous studies (Ratcliff & Rouder, 1998) have demonstrated that between-trial variability in drift rate and starting point adequately explain differences in response speeds, where error responses are faster than correct ones at extreme accuracy levels and slower at intermediate levels. The between-trial variability in the starting point, which reflects an individual’s “bias” toward one choice over the other, contributes to “faster error” responses due to this bias. Meanwhile, the LFM attributes fast error responses to variability in the accumulation of information, or more specifically, to the “noise” in the decision process. Noise or bias in human judgment has already been investigated by Kahneman et al. (2021), though their context differs from this study. Exploring how their concepts could improve cognitive and perceptual decision-making models may provide valuable insights, but such an investigation is beyond the scope of the current study.

### Alpha, jumps and decision dynamics

In the DDM, the noise is assumed to be normally distributed (Ratcliff, 1978), implying that deviations from the drift (the average rate of evidence accumulation) are symmetrically distributed and diminish rapidly as they move away from the mean. On the other hand, in the LFM, the noise assumes a heavy-tailed distribution, allowing for infrequent but large deviations from the drift. We mentioned how Voss et al. (2019) suggest that these jumps in the process can enhance the decision process in “certain” situations.

Further bolstering this hypothesis, *t* test analysis from the current study corroborates their hypothesis, indicating that the impact of practice from session one to session two augmented information processing, evident through increased drift rates and reduced motor and encoding times, as reflected by shorter non-decision times. With the lowered threshold, participants adopted a more liberal decision-making criterion in the second session. Additionally, the subtractive effect on alpha suggests that a lower alpha (indicating more frequent jumps) correlates with improved performance. But about shifting in their hypothesis, we have another idea. They admit that the idea that jumps in the accumulation processes represents an effective switching of attentional resources is rather speculative. We propose “Dual Process Theory” as the guiding logic to investigate what alpha might represent in the accumulation processes. In this theory, dual process approaches describe cognitive performance as a product of two interacting systems (Kahneman & Frederick, 2002; Kahneman, 2011). System 1 and System 2 represent the core components of Dual Process models; the former is fast, intuitive, and emotional, while the latter is slower, more deliberative, and logical. While Dual Process models have traditionally been applied to perceptual tasks such as detection and attention (Schneider & Shiffrin, 1977), the introduction of Adele Diederich’s dynamic-stochastic dual process model provides a mathematical framework for detailing and predicting the interactions between these systems (Diederich, 2024). In her research, Diederich explored two distinct processing architectures: one is a serial process that begins with System 1 and transitions to System 2, and the other is a quasi-parallel process in which both systems operate in “parallel” with crosstalk. Her ’Parallel Dynamic-Stochastic Dual Process Model’ suggests a model that assumes rapid switching between the two systems, resulting in a form of “pseudo-parallel” processing with dependencies.

The intrinsic reliability of the alpha parameter, its strong positive correlation with mean error response times, and its predictability from the first quartile of both correct and error response times, along with a positive relationship with non-decision time and accuracy, lead us to propose that the jumps observed in the decision process under the LFM could be indicative of pseudo-parallel processing within the dual process framework. The alpha parameter, in particular, may capture the extent to which an individual relies on either system, providing a quantitative measure of this dynamic interaction. Those with lower alpha values tend to rely more on System 1, which is fast, deliberate, intuitive, biased, and also more prone to errors.

When System 1 is in charge, it enables faster responses and, due to its error-prone nature, when errors occur, they are typically among these fast responses. Alpha’s positive relationship with accuracy further supports the predominance of System 1 in lower alpha. Additionally, a lower allocation of non-decision time for cognitive processing of stimuli also hints that System 1 is more influential.

Dual process theory can also explain the phenomenon known as ’jumping to conclusions. As Wieschen et al. (2020) states: “*If* a sudden jump in accumulated evidence directly reaches the decision threshold, it resembles jumping to conclusions.” While current methodologies may not allow us to precisely determine which processes end with these sudden jumps, exploring ’jumping to conclusions’ through the Dual Process framework, guided by the value of the alpha parameter, might prove more fruitful. However, these conclusions are tentative and must be substantiated through additional rigorous research. Future studies should aim to refine the methodologies and expand the empirical bases to confirm or refute the hypothesized mechanisms of alpha’s influence and its implications for cognitive processing within different decision-making paradigms.

## Conclusion

This study provided the first comprehensive examination of the reliability of the LFM parameters, with particular emphasis on the stability parameter alpha. Our results suggest that the LFM parameters, including alpha, capture stable inter-individual differences in decision-making and may hold psychological significance. By applying the LFM with alpha as a free parameter, we imply that unobservable internal and external factors that we call LPs influencing outcomes are distinct for each individual and are captured through the noise distribution encoded by the alpha parameter. These findings highlight the need for further investigation into the psychological relevance of alpha and for the development of a theoretical framework that clarifies its role in cognitive modeling and empirical research.

Our analysis showed that alpha has a strong positive relationship with mean reaction times of error responses, indicating its critical role in explaining fast error responses. Additionally, the examination of the predicted decision-time distribution revealed that lower alpha values correspond to shorter response times in the initial quartile of both correct and error responses, emphasizing the dynamics of rapid decision-making.

Our study underscores alpha’s pivotal role in characterizing response time distributions and decision-making variability. Alpha’s reliability and its role in reflecting different aspects of cognitive processing highlight the need for a theoretical framework to enhance the interpretative power of the LFM. Future research should focus on developing this framework and expanding empirical bases to explore the implications of alpha in cognitive processing within different decision-making paradigms. An important direction for future work is the development of a coherent theoretical framework, along with formal tests to determine whether the model’s parameters satisfy selective influence assumptions – an essential step toward validating cognitive interpretations (e.g., (Dutilh et al., 2019; Jones & Dzhafarov, 2014; Smith & Lilburn, 2020)).

Additionally, to start with a theoretical framework, we proposed that the jumps may reflect pseudo-parallel processing (Diederich, 2024) within the dual process framework (Kahneman & Frederick, 2002), potentially indicating the extent to which an individual relies on fast, intuitive (System 1) or slower, deliberative (System 2). This dual process perspective could provide a deeper understanding of decision-making dynamics and the role of alpha in these cognitive mechanisms. However, this hypothesis is speculative and requires further rigorous research to substantiate. Future studies should aim to refine methodologies and confirm or refute the hypothesized mechanisms of alpha’s influence on cognitive processing.

## Supplementary Information

Below is the link to the electronic supplementary material.

Supplementary file 1 (pdf 11035 KB)

## Data Availability

The datasets used in this study were obtained from previously published research. The second dataset, Yap et al. (2012), is publicly accessible through the English Lexical Project (ELP) Website as well as here https://osf.io/n63s2/. For the other datasets, data are not publicly available but were shared with us upon request by the original authors following a review of our study requirements. Researchers interested in accessing these datasets can contact the original authors directly: Lerche and Voss (2017); Schubert et al. (2016). No new data were generated specifically for this study. To comply with the transparency and openness guidelines of the journal, we have generated and thoroughly documented synthetic versions of the restricted datasets. These synthetic datasets, along with all analysis and modeling codes, are openly accessible through our OSF repository: https://osf.io/qmkgn/. These synthetic datasets are designed to replicate the structure and characteristics of the original data while maintaining the confidentiality and ethical standards associated with the restricted datasets.

## Footnotes

1 This assumption allowed us to adopt familiar terminology (e.g., drift rate, threshold, cognitive processes) from the DDM to describe the LFM parameters, purely for the sake of clarity and continuity. We explicitly acknowledge that whether these parameters will retain the same theoretical meaning within a future formal framework for the LFM remains an open question. It is also possible that the LFM, despite its flexibility, may not ultimately prove suitable for uncovering the underlying cognitive processes.

2 Although our priors follow Wagenmakers et al. (2007), more recent reviews synthesizing findings from dozens of DDM studies have since appeared (e.g., Matzke and Wagenmakers (2009); Tran et al. (2021)), and readers may wish to consult these for updated guidance on plausible parameter ranges.

3 As previously noted, this assumption refers to the provisional use of DDM terminology for interpretive clarity, while acknowledging the lack of a formal theoretical mapping between LFM parameters and cognitive processes.

4 Note that the lack of statistically significant test–retest reliability in a task does not imply the index is meaningless; it may reflect occasion-specific (state) variance rather than stable (trait) variance. Advanced methods, such as Latent State-Trait analysis (Steyer et al., 2012), can disentangle trait, state, and error components. However, implementing such analyses requires splitting the data, which is currently impractical due to limitations in our estimation method when trial counts are low. Future work may address this through improved estimation procedures.

## Code availability

The code and other relevant materials used in this study are available on the Open Science Framework (OSF) at https://osf.io/qmkgn/.
