# Supplementary material for: Investigating the potential psychological significance of the alpha parameter in the Lévy flight model of decision making: A reliability analysis approach
Source: Behav Res Methods. 2025 Aug 26;57(10):269. doi: 10.3758/s13428-025-02784-2 (PMC12380952; doi:10.3758/s13428-025-02784-2)
Supplement: Supplementary file 1 — (pdf 11035 KB) [file 13428_2025_2784_MOESM1_ESM.pdf]

Supplementary material: Investigating the Potential Psychological Significance of the Alpha Parameter in the Lévy Flight Model of Decision Making: A Reliability Analysis Approach

Mehdi Ebrahimi Mehr[1] and Jamal Amani Rad[2*]

[1]Institute for Cognitive and Brain Sciences, Shahid Beheshti University, Tehran, Iran.
[2]Choice Modelling Centre & Institute for Transport Studies, University of Leeds, Leeds LS2 9JT, UK.

*Corresponding author(s). E-mail(s): j.amanirad@leeds.ac.uk;
Contributing authors: mehdiebrahimimehr.cs@gmail.com;
m.ebrahimimehr@mail.sbu.ac.ir;

Appendix A Accuracy of Parameter Recovery in BayesFlow Method

This appendix presents a comprehensive evaluation of BayesFlow's performance across various trial sizes (N) specifically for the LFM. The analysis covers eight models, each representing distinct tasks and models, and examines the performance metrics over different trial numbers (N). Note that the x-axis values in Figure A.1 indicate the number of trials "per" condition, meaning the total trials are doubled for two conditions.

To ensure replicability and transparency, we detail the prior distributions used to generate simulated training data for the neural networks. In general, the drift rate was sampled from $\mathcal{U}(0, 5)$, the starting point ratio z_r from $\mathcal{U}(0.3, 0.7)$, non-decision time ndt from $\mathcal{U}(0.1, 0.7)$, and across-trial variability in non-decision time s_{ndt} from $\mathcal{U}(0, 0.3)$. For tasks with two conditions, the second drift rate was drawn from $\mathcal{U}(-5, 0)$. For the APT task with four conditions, drift rates were drawn as $v_1 \sim \mathcal{U}(0, 5)$, $v_2 \sim \mathcal{U}(0, 5)$, $v_3 \sim \mathcal{U}(-5, 0)$, and $v_4 \sim \mathcal{U}(-5, 0)$. The non-decision times for each condition in APT were sampled independently: $ndt_1 \sim \mathcal{U}(0.1, 0.7)$, $ndt_2 \sim \mathcal{U}(0.1, 0.7)$, $ndt_3 \sim \mathcal{U}(0.1, 0.7)$,

and $ndt_4 \sim \mathcal{U}(0.1, 0.7)$. The stability parameters α were drawn as $\alpha \sim \mathcal{U}(1, 2)$. For lower α values, the process becomes increasingly unstable, and convergence to the mean (zero in our simulation) requires substantially more samples. When $\alpha < 2$, the variance becomes infinite, and convergence of the mean increasingly requires more samples, making parameter recovery unreliable without sufficiently large samples (see pp. 48–49 (Nolan, 2020)). Although decreasing time steps in the simulation function increases computational resolution, it also significantly raises computational cost. To balance reliability and feasibility, we set the minimum α to 1. The recovered alpha values confirmed that this range were chosen appropriately for the datasets used in this study.

Figure A.1 showcases the Normalized Root Mean Square Error (NRMSE) on the left panels and the R^2 values on the right panels for each model as a function of the number of trials.

The results indicate acceptable recovery precision of all LFM parameters, evidenced by decreasing NRMSE values with an increasing number of trials. For the maximum trial numbers, NRMSE values range from 0.025 to 0.15 in Model 1 and from 0.015 to 0.07 in Model 2, demonstrating high recovery accuracy. Correspondingly, the R^2 values improve with more trials, achieving values between 0.7 and 0.95, with Model 2 exhibiting slightly better performance. This trend reflects enhanced model fit and predictive accuracy, with consistent improvement as more trials are added.

The parameter α presents the greatest estimation challenge, requiring more trials for accurate recovery. In contrast, other parameters can be estimated with high precision. The figure confirms that increasing the number of trials enhances parameter recovery precision, as evidenced by increasing R^2 values. This pattern is consistent across all models and parameters, underscoring the robustness and reliability of the BayesFlow method for parameter estimation.

This information is crucial for designing future studies, enabling researchers to determine the necessary number of trials for accurate parameter recovery.

In summary, the analysis demonstrates that BayesFlow delivers acceptable parameter recovery, with performance improving as the number of trials increases.

To further illustrate BayesFlow's performance, we include an analysis of parameter recovery for the maximum number of trials for each model. Figure A.2 presents scatter plots for each parameter, showing the relationship between the ground truth (x-axes) and the estimated values (y-axes). The plots highlight the accuracy of parameter recovery, with each subplot corresponding to a different model and parameter combination. High correlation and alignment along the diagonal indicate reasonable parameter recovery, demonstrating the efficacy of BayesFlow for the LFM at the large trial sizes.

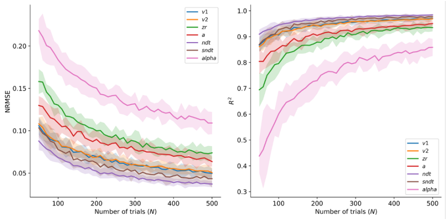

(a) LDT and RMT Model 1

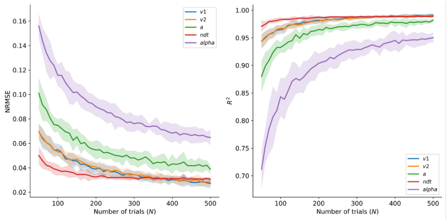

(b) LDT and RMT Model 2

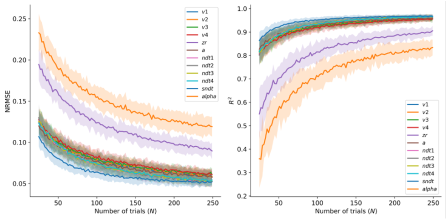

(c) APT Model 1

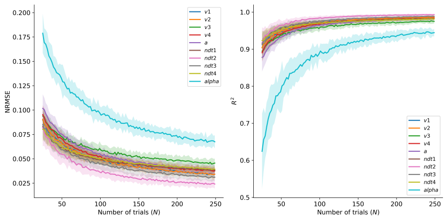

(d) APT Model 2

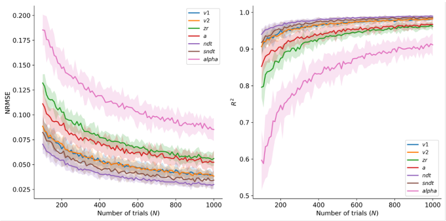

(e) ELP Model 1

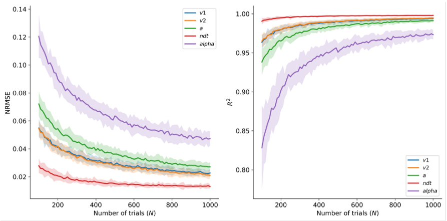

(f) ELP Model 2

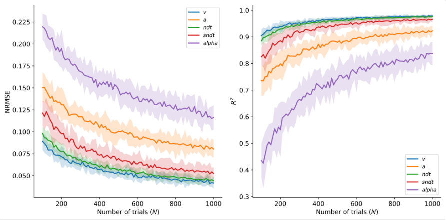

(g) SCHUBERT Study Model 1

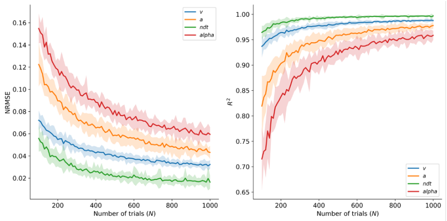

(h) SCHUBERT Study Model 2

Fig. A.1: BayesFlow's performance over all trial numbers (N) for the two models using the LFM. The left panels show the Normalized Root Mean Square Error (NRMSE) and the right panels show the R^2 values as functions of the number of trials. Each x-axis value represents trials per condition; therefore, for two conditions, the total number of trials is double the number indicated on the axis. The results indicate a trend of decreasing NRMSE and increasing R^2 values with the increasing number of trials, demonstrating improved parameter recovery accuracy and model fit with more trials.

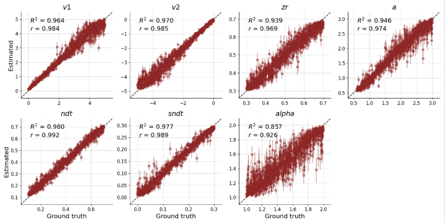

(a) Parameter recovery (T = 1000) LDT and RMT Model 1

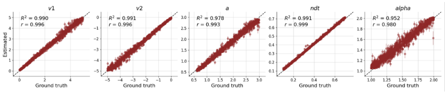

(b) Parameter recovery (T = 1000) LDT and RMT Model 2

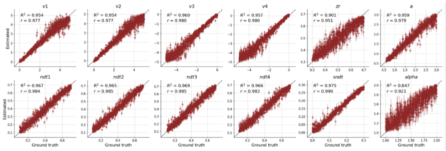

(c) Parameter recovery (T = 1000) APT Model 1

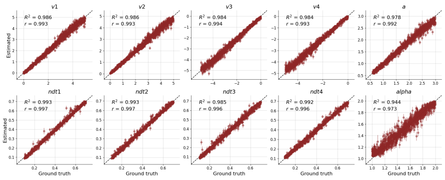

(d) Parameter recovery (T = 1000) APT Model 2

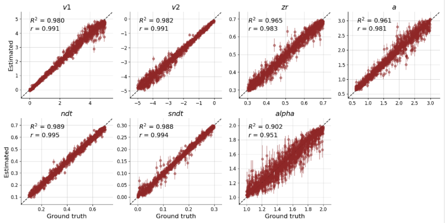

(e) Parameter recovery (T = 2000) ELP Model 1

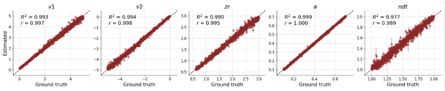

(f) Parameter recovery (T = 2000) ELP Model 2

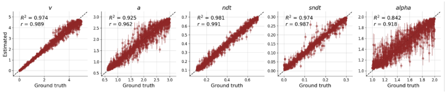

(g) Parameter recovery (T = 1000) SCHUBERT Study Model 1

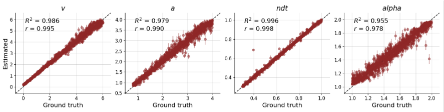

(h) Parameter recovery (T = 1000) SCHUBERT Study Model 2

Fig. A.2: Parameter recovery for all models at the maximum number of trials (T). The x-axes represent the ground truth values, and the y-axes represent the estimated values. The scatter plots demonstrate a high degree of correlation between the ground truth and estimated values, indicated by the points aligning closely along the diagonal, thus confirming the robustness of BayesFlow in accurately recovering parameters for the LFM at the specified trial sizes.

Appendix B Correlation Tables for LFM Parameters Across Measurement Points (ECTs, Schubert study)

(a) Correlations between Alpha parameters at the first and second laboratory session (ECTs, Schubert Study). Maximum number of outlier is 3.

task	0 bit	1 bit	2 bit	PI	NI	S1	S3	S5	0 bit_2	1 bit_2	2 bit_2	PI_2	NI_2	S1_2	S3_2	S5_2
0-bit	1.0***															
1-bit	0.04	1.0***														
2-bit	-0.16	0.07	1.0***													
PI	-0.02	0.1	0.11	1.0***												
NI	-0.06	0.11	0.23**	0.38***	1.0***											
S1	-0.0	0.07	-0.05	0.06	0.06	1.0***										
S3	0.21*	0.04	0.15	0.24**	0.18*	0.04	1.0***									
S5	0.08	0.06	-0.02	0.22*	0.33***	0.06	0.17	1.0***								
0-bit_2	0.68***	-0.03	-0.09	-0.09	-0.07	0.05	0.02	0.08	1.0***							
1-bit_2	0.22*	0.47***	-0.08	0.11	0.07	0.03	-0.02	0.03	0.1	1.0***						
2-bit_2	-0.11	0.09	0.55***	0.02	0.17	0.08	0.18	0.07	0.04	-0.14	1.0***					
PI_2	-0.02	-0.04	0.13	0.43***	0.19*	0.13	0.06	0.07	0.05	-0.04	0.04	1.0***				
NI_2	-0.1	-0.02	0.16	0.28**	0.4***	-0.07	-0.1	0.09	0.0	0.01	0.12	0.38***	1.0***			
S1_2	0.18	-0.09	0.08	0.22*	0.09	0.26**	0.09	-0.01	0.17	0.1	0.1	0.1	0.05	1.0***		
S3_2	0.03	-0.01	0.13	0.3**	0.33***	0.11	0.28**	0.36***	0.02	0.04	0.04	0.18	0.25**	0.25**	1.0***	
S5_2	0.11	-0.15	0.02	0.2*	0.07	0.18	0.15	0.11	0.14	-0.08	0.04	0.13	0.2*	0.12	0.19*	1.0***

(b) Correlations between drift rate parameters at the first and second laboratory session (ECTs, Schubert Study). Maximum number of outlier is 3.

	Session 1								Session 2							
task	0 bit	1 bit	2 bit	PI	NI	S1	S3	S5	0 bit_2	1 bit_2	2 bit_2	PI_2	NI_2	S1_2	S3_2	S5_2
0-bit	1.0***															
1-bit	0.48***	1.0***														
2-bit	0.21*	0.54***	1.0***													
PI	0.36***	0.47***	0.41***	1.0***												
NI	0.32***	0.4***	0.45***	0.65***	1.0***											
S1	0.24**	0.48***	0.44***	0.51***	0.5***	1.0***										
S3	0.28**	0.39***	0.38***	0.55***	0.44***	0.7***	1.0***									
S5	0.19*	0.25**	0.31***	0.37***	0.45***	0.42***	0.52***	1.0***								
0-bit_2	0.65***	0.44***	0.24*	0.31***	0.32***	0.26**	0.33***	0.16	1.0***							
1-bit_2	0.49***	0.72***	0.36***	0.38***	0.36***	0.38***	0.43***	0.25**	0.55***	1.0***						
2-bit_2	0.38***	0.54***	0.65***	0.47***	0.41***	0.42***	0.42***	0.38***	0.41***	0.53***	1.0***					
PI_2	0.33***	0.52***	0.41***	0.58***	0.59***	0.42***	0.49***	0.32***	0.43***	0.55***	0.57***	1.0***				
NI_2	0.33***	0.5***	0.52***	0.61***	0.79***	0.42***	0.47***	0.44***	0.48***	0.51***	0.56***	0.73***	1.0***			
S1_2	0.3**	0.55***	0.51***	0.53***	0.49***	0.65***	0.52***	0.39***	0.39***	0.53***	0.55***	0.53***	0.53***	1.0***		
S3_2	0.17	0.49***	0.45***	0.49***	0.46***	0.68***	0.75***	0.62***	0.25**	0.47***	0.52***	0.55***	0.54***	0.66***	1.0***	
S5_2	0.24*	0.48***	0.39***	0.43***	0.45***	0.53***	0.59***	0.64***	0.31***	0.44***	0.49***	0.51***	0.58***	0.64***	0.67***	1.0***

(c) Correlations between threshold parameters at the first and second laboratory session (ECTs, Schubert Study). Maximum number of outlier is 4.

task	0 bit	1 bit	2 bit	PI	NI	S1	S3	S5	0 bit_2	1 bit_2	2 bit_2	PI_2	NI_2	S1_2	S3_2	S5_2
0-bit	1.0***															
1-bit	0.42***	1.0***														
2-bit	0.38***	0.55***	1.0***													
PI	0.33***	0.31***	0.37***	1.0***												
NI	0.18*	0.18*	0.23**	0.39***	1.0***											
S1	0.36***	0.54***	0.61***	0.29***	0.19*	1.0***										
S3	0.17*	0.23**	0.43***	0.39***	0.16	0.47***	1.0***									
S5	0.22*	0.22*	0.42***	0.39***	0.09	0.35***	0.56***	1.0***								
0-bit_2	0.61***	0.53***	0.38***	0.29**	0.24*	0.42***	0.06	0.14	1.0***							
1-bit_2	0.58***	0.68***	0.48***	0.5***	0.25**	0.44***	0.24**	0.33***	0.43***	1.0***						
2-bit_2	0.37***	0.49***	0.76***	0.42***	0.24*	0.47***	0.33***	0.45***	0.38***	0.62***	1.0***					
PI_2	0.16	0.31***	0.31***	0.65***	0.33***	0.31***	0.35***	0.32***	0.22*	0.37***	0.43***	1.0***				
NI_2	0.12	0.13	0.31***	0.53***	0.59***	0.11	0.27**	0.27**	0.1	0.27**	0.35***	0.44***	1.0***			
S1_2	0.34***	0.34***	0.63***	0.4***	0.17	0.55***	0.4***	0.44***	0.4***	0.41***	0.57***	0.23*	0.19*	1.0***		
S3_2	0.14	0.32***	0.52***	0.31***	0.21*	0.54***	0.58***	0.46***	0.21*	0.31***	0.5***	0.32***	0.23*	0.57***	1.0***	
S5_2	-0.01	0.05	0.33***	0.18	0.08	0.25**	0.51***	0.5***	-0.06	0.11	0.34***	0.24**	0.2*	0.31***	0.56***	1.0***

1

(d) Correlations between non-decision time parameters at the first and second laboratory session (ECTs, Schubert Study). Maximum number of outlier is 6.

task	0 bit	1 bit	2 bit	PI	NI	S1	S3	S5	0 bit_2	1 bit_2	2 bit_2	PI_2	NI_2	S1_2	S3_2	S5_2
0-bit	1.0***															
1-bit	0.46***	1.0***														
2-bit	0.44***	0.65***	1.0***													
PI	0.15	0.36***	0.26**	1.0***												
NI	0.15	0.29***	0.28***	0.68***	1.0***											
S1	0.16	0.41***	0.19*	0.46***	0.49***	1.0***										
S3	0.27**	0.35***	0.43***	0.41***	0.43***	0.26**	1.0***									
S5	0.25**	0.29***	0.29***	0.54***	0.56***	0.33***	0.71***	1.0***								
0-bit_2	0.59***	0.41***	0.36***	0.25**	0.28**	0.15	0.16	0.26**	1.0***							
1-bit_2	0.42***	0.55***	0.52***	0.31***	0.32***	0.26**	0.32***	0.28**	0.44***	1.0***						
2-bit_2	0.27**	0.46***	0.6***	0.38***	0.45***	0.31***	0.41***	0.25**	0.23*	0.56***	1.0***					
PI_2	0.14	0.35***	0.29**	0.59***	0.49***	0.39***	0.27**	0.33***	0.33***	0.47***	0.47***	1.0***				
NI_2	0.15	0.36***	0.36***	0.71***	0.7***	0.33***	0.34***	0.4***	0.34***	0.34***	0.49***	0.63***	1.0***			
S1_2	0.21*	0.4***	0.45***	0.49***	0.36***	0.5***	0.35***	0.31***	0.37***	0.53***	0.47***	0.5***	0.49***	1.0***		
S3_2	0.19*	0.27**	0.36***	0.5***	0.44***	0.34***	0.73***	0.66***	0.25**	0.16	0.4***	0.32***	0.41***	0.47***	1.0***	
S5_2	0.35***	0.3**	0.36***	0.42***	0.49***	0.25**	0.62***	0.62***	0.39***	0.34***	0.39***	0.39***	0.45***	0.42***	0.66***	1.0***

Table B.1: Correlation Tables for LFM Parameters Across Measurement Points (ECTs, Schubert study)

Appendix C Simulation-Based Calibration (SBC)

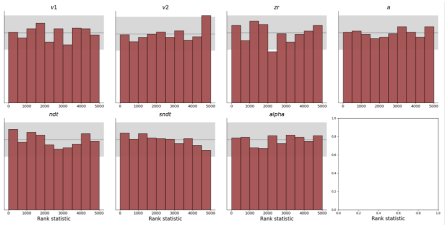

(a) SBC at N = 1000. LDT and RMT Model 1

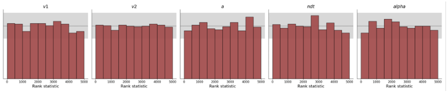

(b) SBC at N = 1000. LDT and RMT Model 2

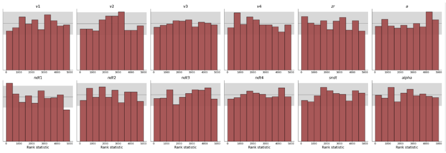

(c) SBC at N = 1000. APT Model 1

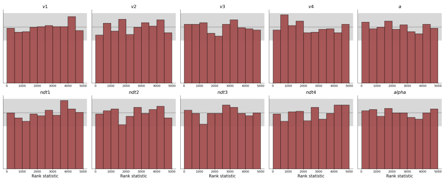

(d) SBC at N = 1000. APT Model 2

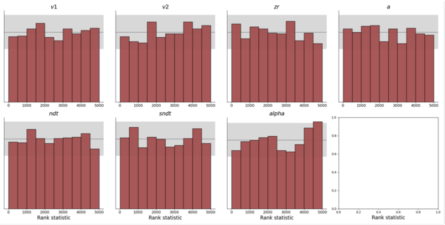

(e) SBC at N = 2000. ELP Model 1

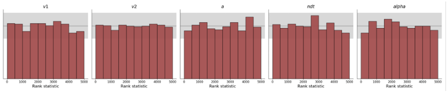

(f) SBC at N = 2000. ELP Model 2

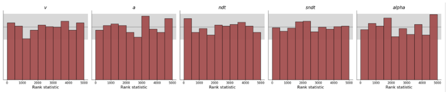

(g) SBC at N = 1000. SCHUBERT Study Model 1

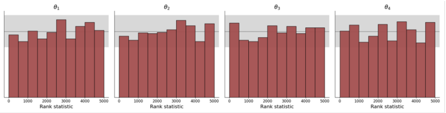

(h) SBC at N = 1000. SCHUBERT Study Model 2

Fig. C.3: The histograms are roughly uniform and lie within the expected range for well-calibrated inference algorithms as indicated by the shaded gray areas. Accordingly, our neural approximator seems to have converged to the intended target.

Appendix D *t*-test Results

This section provides an overview of the *t*-test results for all tasks and both models, aimed at assessing the practice effect from session one to session two. The analysis

focuses on the differences in means, standard deviations, and the statistical significance of these differences. Across various tasks and models, significant differences were observed in key parameters of demanding tasks, such as drift rates, non-decision time, threshold, starting point, and alpha.

Table D.2 presents the t-test results for Model 1 of the LDT and RMT tasks, highlighting significant changes across sessions. Similarly, Table D.3 details the t-test results for Model 2, emphasizing significant differences in parameters from session one to session two.

The t-test results for Model 1 of the APT are summarized in Table D.4, while Table D.5 provides the results for the parameters in Model 2 of the APT.

For the ELP task, Table D.6 (Model 1) and Table D.7 (Model 2) offer a comparison of key parameters such as drift rates, non-decision time, threshold, and alpha, with significant differences observed across sessions.

Finally, the t-test results for the ECTs —Hick, Posner, and Stern—detailed in Tables D.8, D.9, and D.10, respectively, did not show significant practice effects, as expected, given that these are less demanding tasks.

Table D.2: Model 1 t-test Result for LDT and RMT. $v_{\text{total}} = v1 - v2$, $v_{\text{total}} = v1 + v2$

parameter	mean_s1	std_s1	mean_s2	std_s2	T	dof	p-val	CI95%	cohen-d
				**LDT**					
v1	2.1116	0.5032	2.4654	0.6377	-8.4683	103	< 0.0001	[-0.44 -0.27]	0.6159
v2	-2.8177	0.5531	-2.9095	0.6386	2.2204	103	0.0286	[0.01 0.17]	0.1537
zr	0.5324	0.0508	0.4997	0.0586	6.1746	103	< 0.0001	[0.02 0.04]	0.5964
v_{total}	4.9292	0.9157	5.3748	1.1371	-7.9149	103	< 0.0001	[-0.56 -0.33]	0.4316
v_{bias}	-0.7061	0.5288	-0.4441	0.5795	-4.2825	103	< 0.0001	[-0.38 -0.14]	0.4722
a	1.1911	0.2761	1.1197	0.299	3.8253	103	0.0002	[0.03 0.11]	0.2481
ndt	0.4519	0.0386	0.4387	0.046	3.0069	103	0.0033	[0. 0.02]	0.311
sndt	0.0238	0.015	0.0248	0.0176	-0.5792	103	0.5637	[-0. 0.]	0.0575
mean_log_rt	6.5075	0.1151	6.4466	0.12	7.4124	103	< 0.0001	[0.04 0.08]	0.5183
alpha_T	0.2242	0.2151	0.1215	0.2011	5.6254	103	< 0.0001	[0.07 0.14]	0.4933
accuracy_T	0.8602	0.3028	0.6954	0.2426	12.0246	103	< 0.0001	[0.14 0.19]	0.6007
alpha	1.8159	0.1481	1.7616	0.182	3.5389	103	0.0006	[0.02 0.08]	0.3271
				**RMT**					
v1	1.8274	0.8709	2.3838	1.0637	-7.5714	102	< 0.0001	[-0.7 -0.41]	0.5724
v2	-2.0858	0.6125	-2.4658	0.8658	5.5378	102	< 0.0001	[0.24 0.52]	0.5067
zr	0.532	0.0643	0.5361	0.0634	-0.6029	102	0.5479	[-0.02 0.01]	0.0647
v_{total}	3.9133	1.3159	4.8496	1.7657	-8.0862	102	< 0.0001	[-1.17 -0.71]	0.6013
v_{bias}	-0.2584	0.7318	-0.082	0.8027	-2.1378	102	0.0349	[-0.34 -0.01]	0.2296
a	1.3533	0.3212	1.2828	0.3079	2.8054	102	0.006	[0.02 0.12]	0.2242
ndt	0.5846	0.047	0.5275	0.0521	10.9761	102	< 0.0001	[0.05 0.07]	1.1509
sndt	0.0339	0.0224	0.0258	0.0197	4.2416	102	< 0.0001	[0. 0.01]	0.3867
mean_log_rt	6.7637	0.1062	6.6322	0.1043	16.285	102	< 0.0001	[0.12 0.15]	1.2495
alpha_T	0.3969	0.2121	0.1665	0.1634	9.7216	102	< 0.0001	[0.18 0.28]	1.2169
accuracy_T	0.3303	0.1734	0.3966	0.2013	-5.1912	102	< 0.0001	[-0.09 -0.04]	0.3526
alpha	1.8765	0.0698	1.8249	0.1368	3.7772	102	0.0003	[0.02 0.08]	0.4751

Table D.3: Model 2 t-test Result for LDT and RMT. $v_{\text{total}} = v1 - v2$, $v_{\text{total}} = v1 + v2$

parameter	mean_s1	std_s1	mean_s2	std_s2	T	dof	p-val	CI95%	cohen-d
				**LDT**					
v1	2.3983	0.5693	2.6104	0.6674	-5.3923	103	< 0.0001	[-0.29 -0.13]	0.342
v2	-2.4434	0.4832	-2.7043	0.5856	7.5123	103	< 0.0001	[0.19 0.33]	0.4859
a	1.302	0.301	1.2815	0.4015	0.5973	103	0.5516	[-0.05 0.09]	0.0577
v_{total}	4.8417	0.9488	5.3146	1.1666	-7.1845	103	< 0.0001	[-0.6 -0.34]	0.4448
v_{bias}	-0.0451	0.4638	-0.0939	0.4644	1.4234	103	0.1576	[-0.02 0.12]	0.105
ndt	0.4109	0.0526	0.3923	0.0556	3.3366	103	0.0012	[0.01 0.03]	0.3438
alpha	1.7197	0.1971	1.6578	0.2025	4.0406	103	0.0001	[0.03 0.09]	0.3094
mean_log_rt	6.5075	0.1151	6.4466	0.12	7.4124	103	< 0.0001	[0.04 0.08]	0.5183
alpha_T	0.0597	0.2226	-0.0196	0.209	4.7955	103	< 0.0001	[0.05 0.11]	0.3673
accuracy_T	0.8602	0.3028	0.6954	0.2426	12.0246	103	< 0.0001	[0.14 0.19]	0.6007
				**RMT**					
v1	2.0369	0.921	2.6332	1.1209	-8.2157	102	< 0.0001	[-0.74 -0.45]	0.5813
v2	-1.6937	0.6142	-2.0685	0.8795	5.7436	102	< 0.0001	[0.25 0.5]	0.4942
a	1.4062	0.3529	1.3575	0.3585	1.4962	102	0.1377	[-0.02 0.11]	0.137
v_{total}	3.7306	1.4234	4.7018	1.874	-7.9283	102	< 0.0001	[-1.21 -0.73]	0.5836
v_{bias}	0.3432	0.6519	0.5647	0.7401	-3.4795	102	0.0007	[-0.35 -0.1]	0.3176
ndt	0.541	0.0465	0.4859	0.0602	9.0926	102	< 0.0001	[0.04 0.07]	1.0264
alpha	1.818	0.149	1.7624	0.1859	2.9855	102	0.0035	[0.02 0.09]	0.3301
mean_log_rt	6.7637	0.1062	6.6322	0.1043	16.285	102	< 0.0001	[0.12 0.15]	1.2495
alpha_T	0.1992	0.2209	0.1202	0.2301	3.4997	102	0.0007	[0.03 0.12]	0.3502
accuracy_T	0.3303	0.1734	0.3966	0.2013	-5.1912	102	< 0.0001	[-0.09 -0.04]	0.3526

Table D.4: Model 1 t-test Result APT. $v_{\text{total}} = (v2{+}v1){-}(v3{+}v4)$, $v_{\text{bias}} = (v2{+}v1){+}(v3{+}v4)$, $v_{\text{w\_priming}} = (v2 - v1)$, $v_{\text{nw\_priming}} = (v4 - v3)$

parameter	mean_s1	std_s1	mean_s2	std_s2	T	dof	p-val	CI95%	cohen-d
v1	2.1883	0.588	2.4916	0.6683	-6.4532	127	< 0.0001	[-0.4 -0.21]	0.4818
v2	1.9537	0.4808	2.2217	0.6018	-7.0454	127	< 0.0001	[-0.34 -0.19]	0.492
v3	-2.0552	0.4826	-2.2871	0.5823	5.8912	127	< 0.0001	[0.15 0.31]	0.4336
v4	-2.1282	0.5304	-2.4216	0.6272	6.6119	127	< 0.0001	[0.21 0.38]	0.5052
zr	0.5316	0.0615	0.5178	0.0625	2.4003	127	0.0178	[0. 0.03]	0.2233
v_{total}	8.3254	1.7992	9.422	2.1791	-9.3766	127	< 0.0001	[-1.33 -0.87]	0.5488
v_{bias}	-0.0414	0.8669	0.0045	0.9527	-0.4856	127	0.6281	[-0.23 0.14]	0.0504
$v_{\text{w\_priming}}$	0.2346	0.3437	0.2699	0.352	-0.8969	127	0.3715	[-0.11 0.04]	0.1014
$v_{\text{nw\_priming}}$	-0.073	0.2643	-0.1345	0.3613	1.5923	127	0.1138	[-0.01 0.14]	0.1945
a	1.3054	0.341	1.1545	0.3068	8.7073	127	< 0.0001	[0.12 0.19]	0.4654
ndt1	0.51	0.0481	0.5006	0.0434	2.6518	127	0.009	[0. 0.02]	0.2061
ndt2	0.516	0.041	0.5061	0.0369	2.965	127	0.0036	[0. 0.02]	0.2549
ndt3	0.5643	0.0515	0.5411	0.0441	5.6501	127	< 0.0001	[0.02 0.03]	0.4824
ndt4	0.5558	0.0504	0.5351	0.0427	5.0124	127	< 0.0001	[0.01 0.03]	0.4441
sndt	0.0128	0.0117	0.0121	0.0148	0.6264	127	0.5322	[-0. 0.]	0.0539
alpha	1.7941	0.1837	1.8187	0.1474	-1.858	127	0.0655	[-0.05 0.]	0.1482
accuracy	0.9341	0.0419	0.9338	0.0499	0.1232	127	0.9022	[-0. 0.]	0.0061
mean_log_rt	6.6997	0.1168	6.6038	0.1066	15.8816	127	< 0.0001	[0.08 0.11]	0.8574
alpha_T	0.1866	0.2274	0.2124	0.214	-1.6192	127	0.1079	[-0.06 0.01]	0.1167
accuracy_T	0.5651	0.1976	0.6786	0.2747	-8.5198	127	< 0.0001	[-0.14 -0.09]	0.4741

Table D.5: Model 2 t-test Result APT.$v_{\text{total}} = (v2{+}v1){-}(v3{+}v4)$, $v_{\text{bias}} = (v2{+}v1){+}(v3{+}v4)$, $v_{\text{w\_priming}} = (v2 - v1)$, $v_{\text{nw\_priming}} = (v4 - v3)$

parameter	mean_s1	std_s1	mean_s2	std_s2	T	dof	p-val	CI95%	cohen-d
v1	2.1951	0.5004	2.3903	0.613	-4.9607	127	< 0.0001	[-0.27 -0.12]	0.3489
v2	2.0235	0.4355	2.2222	0.5592	-5.8193	127	< 0.0001	[-0.27 -0.13]	0.3965
v3	-2.1175	0.5186	-2.3421	0.612	5.7873	127	< 0.0001	[0.15 0.3]	0.3959
v4	-2.1191	0.5013	-2.386	0.5688	6.8928	127	< 0.0001	[0.19 0.34]	0.4978
a	1.3903	0.3302	1.2257	0.3047	9.3416	127	< 0.0001	[0.13 0.2]	0.5181
v_{total}	8.4551	1.7639	9.3405	2.1554	-7.4503	127	< 0.0001	[-1.12 -0.65]	0.4496
v_{total}	-0.018	0.6431	-0.1156	0.6921	1.5796	127	0.1167	[-0.02 0.22]	0.1461
$v_{\text{w\_priming}}$	0.1716	0.3068	0.1681	0.3116	0.1002	127	0.9204	[-0.07 0.07]	0.0112
$v_{\text{nw\_priming}}$	-0.0016	0.2532	-0.0439	0.3372	1.1872	127	0.2374	[-0.03 0.11]	0.1419
ndt1	0.4661	0.0489	0.4582	0.0485	1.9334	127	0.0554	[-0. 0.02]	0.1607
ndt2	0.5009	0.0498	0.494	0.0422	1.7769	127	0.078	[-0. 0.01]	0.149
ndt3	0.5583	0.0521	0.53	0.0452	7.6518	127	< 0.0001	[0.02 0.04]	0.5811
ndt4	0.5388	0.0536	0.5156	0.0459	5.4043	127	< 0.0001	[0.01 0.03]	0.465
alpha	1.7391	0.2059	1.7322	0.1962	0.517	127	0.6061	[-0.02 0.03]	0.0346
accuracy	0.9341	0.0419	0.9338	0.0499	0.1232	127	0.9022	[-0. 0.]	0.0061
mean_log_rt	6.6997	0.1168	6.6038	0.1066	15.8816	127	< 0.0001	[0.08 0.11]	0.8574
alpha_T	0.0972	0.2311	0.0793	0.2248	1.1328	127	0.2594	[-0.01 0.05]	0.0782
accuracy_T	0.5651	0.1976	0.6786	0.2747	-8.5198	127	< 0.0001	[-0.14 -0.09]	0.4741

Table D.6: Model 1 t-test Result ELP. v_total = v2 - v1, v_bias = v1 + v2

parameter	mean_s1	std_s1	mean_s2	std_s2	T	dof	p-val	CI95%	cohen-d
v1	1.2971	0.4843	1.3397	0.5037	-5.7717	805	< 0.0001	[-0.06 -0.03]	0.0863
v2	-1.7385	0.5871	-1.7764	0.6107	3.9634	805	0.0001	[0.02 0.06]	0.0633
v_total = v2 - v1	3.0355	1.0299	3.1161	1.068	-5.8437	805	< 0.0001	[-0.11 -0.05]	0.0768
v_bias = v1 + v2	-0.4414	0.3126	-0.4367	0.3355	-0.4676	805	0.6402	[-0.02 0.02]	0.0146
zr	0.5942	0.0515	0.5917	0.0545	1.6479	805	0.0998	[-0. 0.01]	0.0476
a	1.2286	0.3479	1.2018	0.3441	3.7139	805	0.0002	[0.01 0.04]	0.0774
ndt	0.4847	0.0818	0.4679	0.0842	6.5478	805	< 0.0001	[0.01 0.02]	0.2024
sndt	0.0738	0.0411	0.0669	0.0456	4.5912	805	< 0.0001	[0. 0.01]	0.1589
alpha	1.8349	0.2168	1.8167	0.2136	2.6783	805	0.0076	[0. 0.03]	0.0846
accuracy	0.8608	0.0667	0.8626	0.0701	-1.6698	805	0.0953	[-0. 0.]	0.0257
mean_log_rt	6.6452	0.1987	6.6063	0.2039	9.2286	805	< 0.0001	[0.03 0.05]	0.1932
alpha_T	0.2491	0.2816	0.2067	0.2677	5.0196	805	< 0.0001	[0.03 0.06]	0.1543
accuracy_T	0.2602	0.1378	0.2788	0.151	-8.9455	805	< 0.0001	[-0.02 -0.01]	0.1289

Table D.7: Model 2 t-test Result ELP. $v_{\text{total}} = v2 - v1$, $v_{\text{bias}} = v1 + v2$

parameter	mean_s1	std_s1	mean_s2	std_s2	T	dof	p-val	CI95%	cohen-d
v1	1.3878	0.5282	1.4375	0.5581	-5.6522	805	< 0.0001	[-0.07 -0.03]	0.0915
v2	-1.2928	0.4387	-1.3375	0.4553	5.2491	805	< 0.0001	[0.03 0.06]	0.0999
v_{total}	2.6806	0.9392	2.775	0.9829	-5.9108	805	< 0.0001	[-0.13 -0.06]	0.0982
v_{bias}	0.095	0.2464	0.1	0.2672	-0.7574	805	0.449	[-0.02 0.01]	0.0197
a	1.3585	0.4179	1.3287	0.4515	2.3887	805	0.0171	[0.01 0.05]	0.0685
ndt	0.398	0.088	0.387	0.0904	4.2958	805	< 0.0001	[0.01 0.02]	0.1231
alpha	1.5455	0.2333	1.5536	0.2306	-1.2625	805	0.2071	[-0.02 0.]	0.0349
accuracy	0.8608	0.0667	0.8626	0.0701	-1.6698	805	0.0953	[-0. 0.]	0.0257
mean_log_rt	6.6452	0.1987	6.6063	0.2039	9.2286	805	< 0.0001	[0.03 0.05]	0.1932
alpha_T	-0.1624	0.269	-0.156	0.2734	-0.8376	805	0.4025	[-0.02 0.01]	0.0236
accuracy_T	0.2602	0.1378	0.2788	0.151	-8.9455	805	< 0.0001	[-0.02 -0.01]	0.1289

Table D.8: Model 2 t-test Result Hick Tasks ALL 0-bit, 1-bit and 2-bit

parameter	mean_s1	std_s1	mean_s2	std_s2	T	p-val	CI95%	cohen-d	condition
					**0-bit**				
v	4.8686	0.9594	4.8764	0.9301	-0.0653	0.948	[-0.25 0.23]	0.0083	0-bit
a	0.9913	0.1305	0.9715	0.1261	1.2098	0.2275	[-0.01 0.05]	0.1541	0-bit
ndt	0.2817	0.0061	0.2805	0.0061	1.5441	0.1239	[-0. 0.]	0.1973	0-bit
$alpha$	1.5825	0.3678	1.5185	0.3835	1.3309	0.1845	[-0.03 0.16]	0.1706	0-bit
$alpha\_T$	-0.0119	0.3441	-0.0876	0.361	1.6782	0.0946	[-0.01 0.16]	0.2152	0-bit
					**1-bit**				
v	4.6338	0.9317	4.7171	0.9848	-0.6793	0.4976	[-0.33 0.16]	0.0872	1-bit
a	1.0955	0.2177	1.075	0.1866	0.7963	0.4266	[-0.03 0.07]	0.1004	1-bit
ndt	0.2893	0.0067	0.2889	0.0061	0.5711	0.5684	[-0. 0.]	0.0724	1-bit
$alpha$	1.8201	0.1445	1.8029	0.1688	0.8506	0.3959	[-0.02 0.06]	0.1101	1-bit
alpha_T	0.1839	0.1844	0.1606	0.2015	0.941	0.3477	[-0.03 0.07]	0.1211	1-bit
					**2-bit**				
v	3.9974	0.8294	4.0939	0.8822	-0.8801	0.3797	[-0.31 0.12]	0.113	2-bit
a	1.3958	0.4647	1.3175	0.4548	1.335	0.1832	[-0.04 0.19]	0.1702	2-bit
ndt	0.3005	0.0157	0.3001	0.014	0.2529	0.8006	[-0. 0.]	0.032	2-bit
$alpha$	1.7101	0.1438	1.7114	0.1497	-0.069	0.9451	[-0.04 0.04]	0.0088	2-bit
$alpha\_T$	0.0295	0.1666	0.0314	0.1763	-0.0864	0.9312	[-0.05 0.04]	0.0111	2-bit

Table D.9: Model 2 t-test Result Ponser Task Both PI and NI

parameter	mean_s1	std_s1	mean_s2	std_s2	T	p-val	CI95%	cohen-d	condition
					**PI**				
v	4.7734	0.8612	4.6827	1.062	0.7302	0.466	[-0.15 0.34]	0.0946	PI
a	2.4898	0.4638	2.237	0.4413	4.3919	< 0.0001	[0.14 0.37]	0.5574	PI
ndt	0.334	0.0379	0.3336	0.0363	0.0845	0.9327	[-0.01 0.01]	0.0107	PI
$alpha$	1.862	0.0878	1.8677	0.0854	-0.5171	0.6056	[-0.03 0.02]	0.0657	PI
$alpha\_T$	0.3314	0.2215	0.2897	0.1907	1.5938	0.1123	[-0.01 0.09]	0.2006	PI
					**NI**				
v	3.878	0.8916	4.0503	0.9663	-1.4498	0.1485	[-0.41 0.06]	0.1859	NI
a	2.4522	0.4091	2.5571	0.4297	-1.9599	0.0512	[-0.21 0.]	0.2507	NI
ndt	0.3503	0.0397	0.3458	0.0443	0.8331	0.4057	[-0.01 0.02]	0.1071	NI
$alpha$	1.9345	0.0452	1.9247	0.0538	1.5335	0.1266	[-0. 0.02]	0.1981	NI
$alpha\_T$	0.5847	0.2063	0.4302	0.1537	6.7424	< 0.0001	[0.11 0.2]	0.8394	NI

Table D.10: Model 2 t-test Result Strenberg Task ALL S1,S3, and S5

parameter	mean_s1	std_s1	mean_s2	std_s2	T	p-val	CI95%	cohen-d	condition
					**S1**				
v	3.3103	0.9789	3.5229	1.0506	-1.6385	0.1027	[-0.47 0.04]	0.21	S1
a	1.5521	0.497	1.538	0.4559	0.2337	0.8154	[-0.11 0.13]	0.0296	S1
ndt	0.333	0.036	0.3268	0.038	1.2955	0.1964	[-0. 0.02]	0.1658	S1
$alpha$	1.7832	0.1771	1.7977	0.1475	-0.7045	0.4818	[-0.06 0.03]	0.0885	S1
$alpha\_T$	0.1374	0.2223	0.1524	0.2019	-0.5566	0.5783	[-0.07 0.04]	0.0704	S1
					**S3**				
v	3.2653	0.8969	3.4534	0.9729	-1.5724	0.1172	[-0.42 0.05]	0.2017	S3
a	2.0542	0.5969	2.004	0.6527	0.6278	0.5307	[-0.11 0.21]	0.0806	S3
ndt	0.3868	0.0726	0.3787	0.0668	0.9047	0.3665	[-0.01 0.03]	0.1145	S3
$alpha$	1.7122	0.1812	1.7325	0.1838	-0.8724	0.3839	[-0.07 0.03]	0.1113	S3
$alpha\_T$	0.0378	0.2002	0.069	0.2095	-1.1957	0.233	[-0.08 0.02]	0.1529	S3
					**S5**				
v	2.6021	0.6807	2.7354	0.937	-1.2621	0.2083	[-0.34 0.07]	0.1649	S5
a	2.1205	0.6123	2.028	0.6669	1.1303	0.2595	[-0.07 0.25]	0.145	S5
ndt	0.4535	0.1048	0.4315	0.0966	1.7121	0.0881	[-0. 0.05]	0.2167	S5
$alpha$	1.6645	0.1886	1.6827	0.1886	-0.758	0.4492	[-0.07 0.03]	0.0966	S5
$alpha\_T$	-0.014	0.2066	0.0088	0.2036	-0.8736	0.3832	[-0.07 0.03]	0.1112	S5

Appendix E Correlation Among LFM parameters

In this section, we present a detailed analysis of the correlations among the parameters of the LFM across different sessions and models. This analysis is essential for understanding the unique contributions of each parameter and identifying any potential overlaps in the aspects they measure.

Our analysis reveals that most of the LFM parameters capture distinct aspects of the data with minimal overlap, as indicated by the generally low and often insignificant correlation coefficients. However, a notable exception is the stability parameter, alpha, which consistently shows significant correlations with *ndt* and threshold (a) across most datasets.

Figures E.4 to E.11 collectively illustrate the relationships between different LFM parameters, providing a comprehensive overview of how each parameter interacts with others across various conditions and datasets. These figures also show how distributions approximate normality.

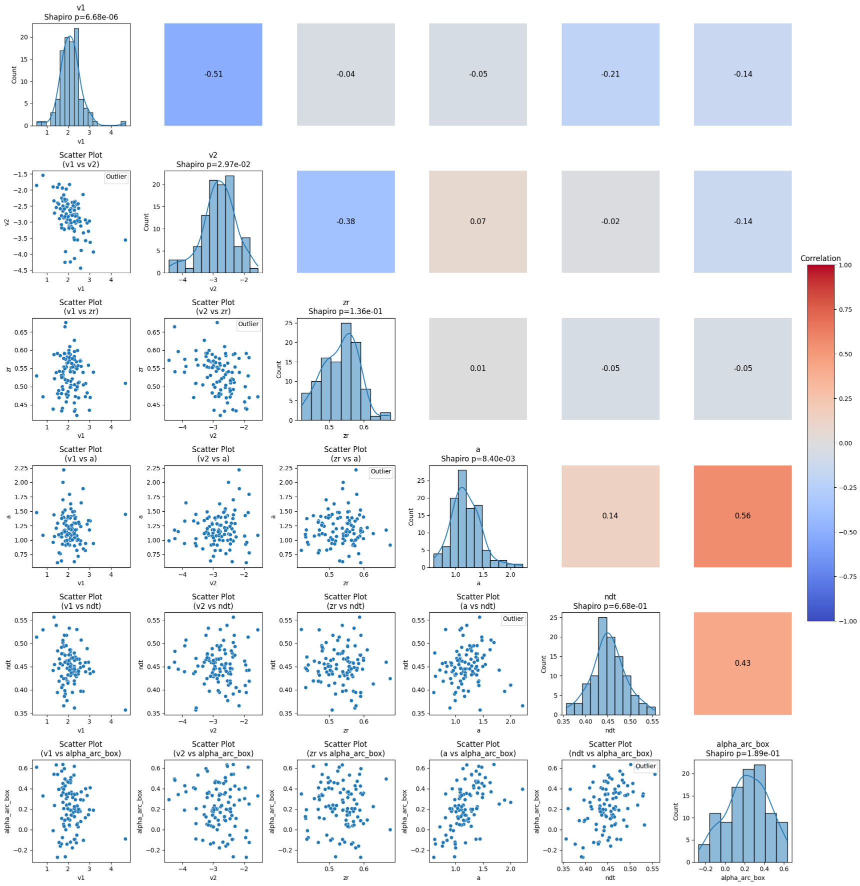

(a) LDT Session 1 Model 1

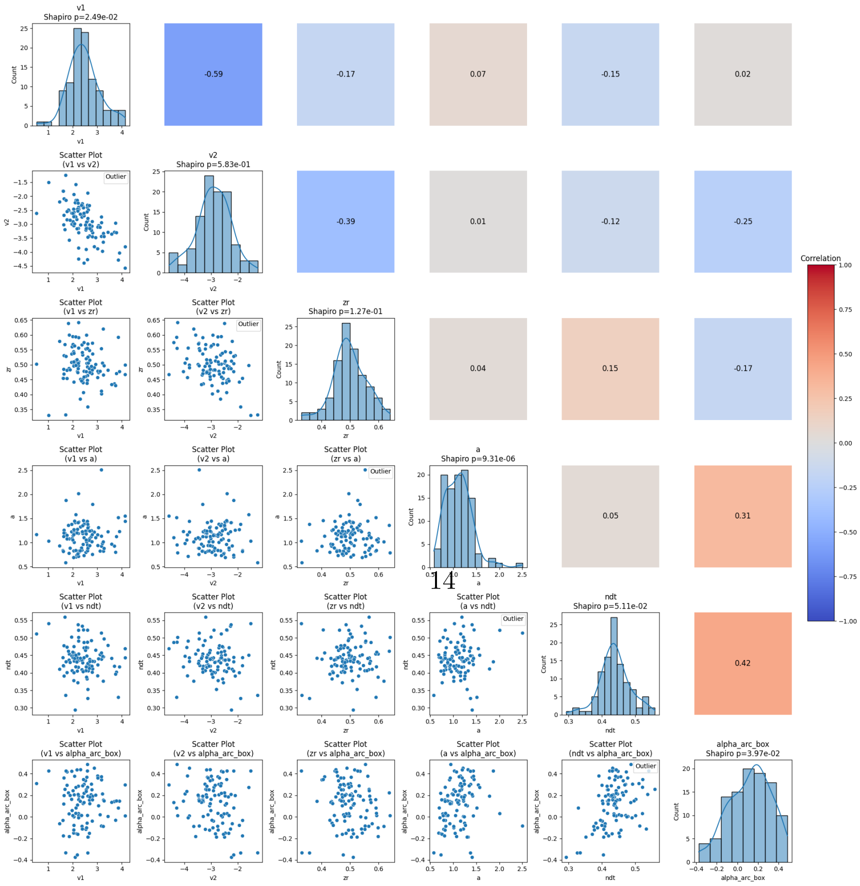

(b) LDT Session 2 Model 1

Fig. E.4: Correlation among LFM parameters for LDT Model 1

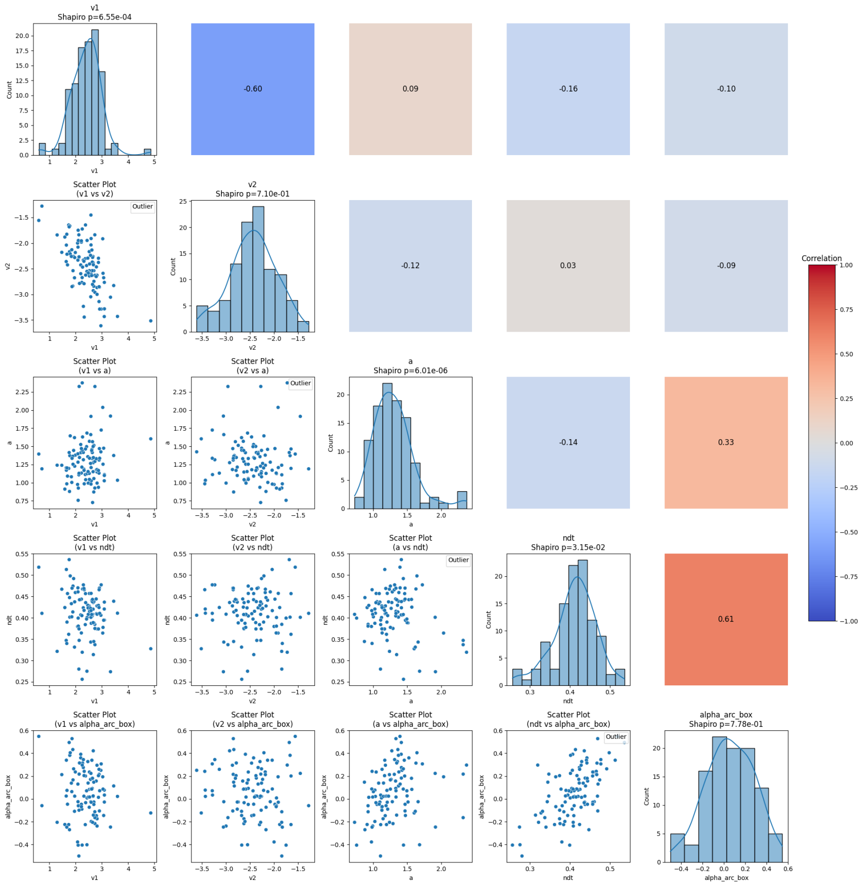

(a) LDT Session 1 Model 2

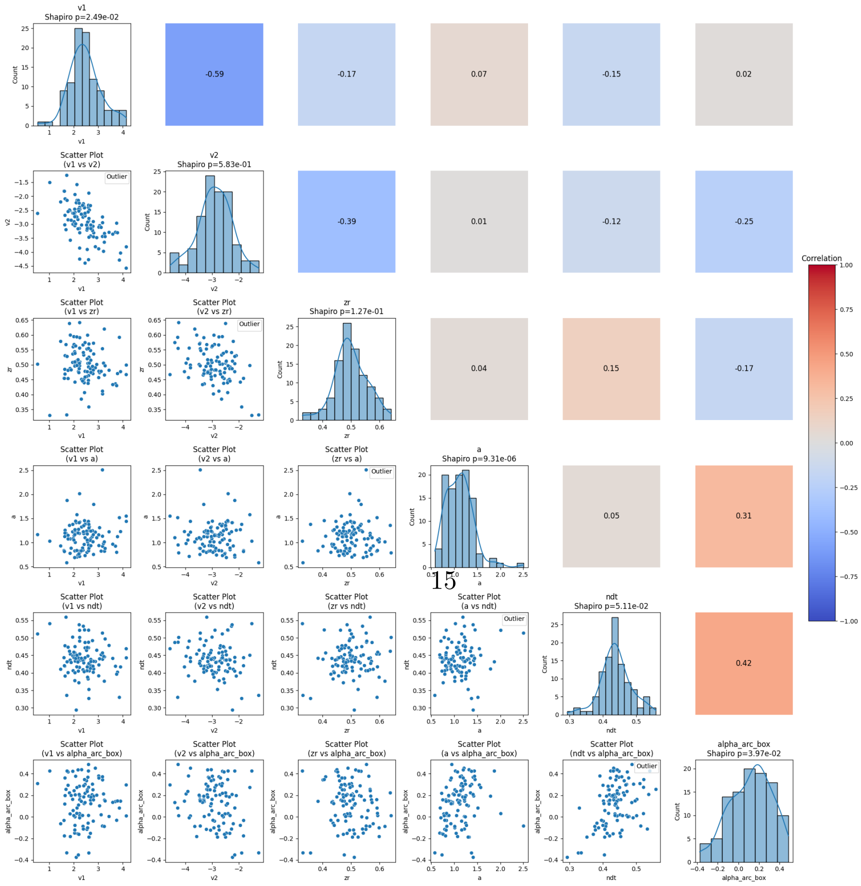

(b) LDT Session 2 Model 2

Fig. E.5: Correlation among LFM parameters for LDT Model 2

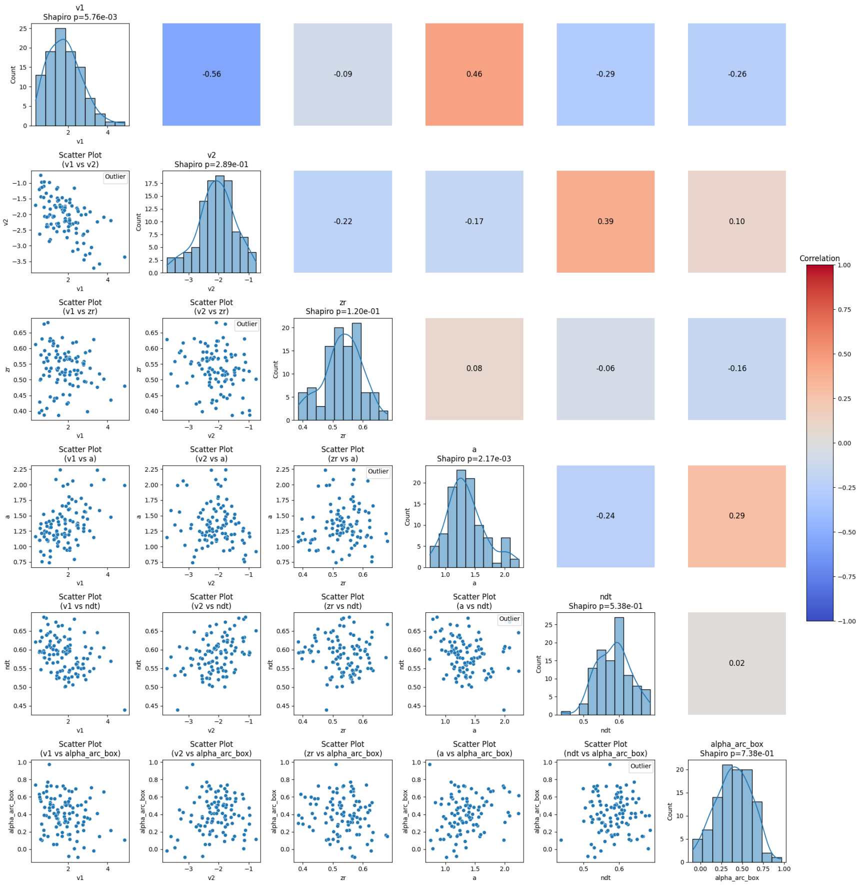

(a) RMT Session 1 Model 1

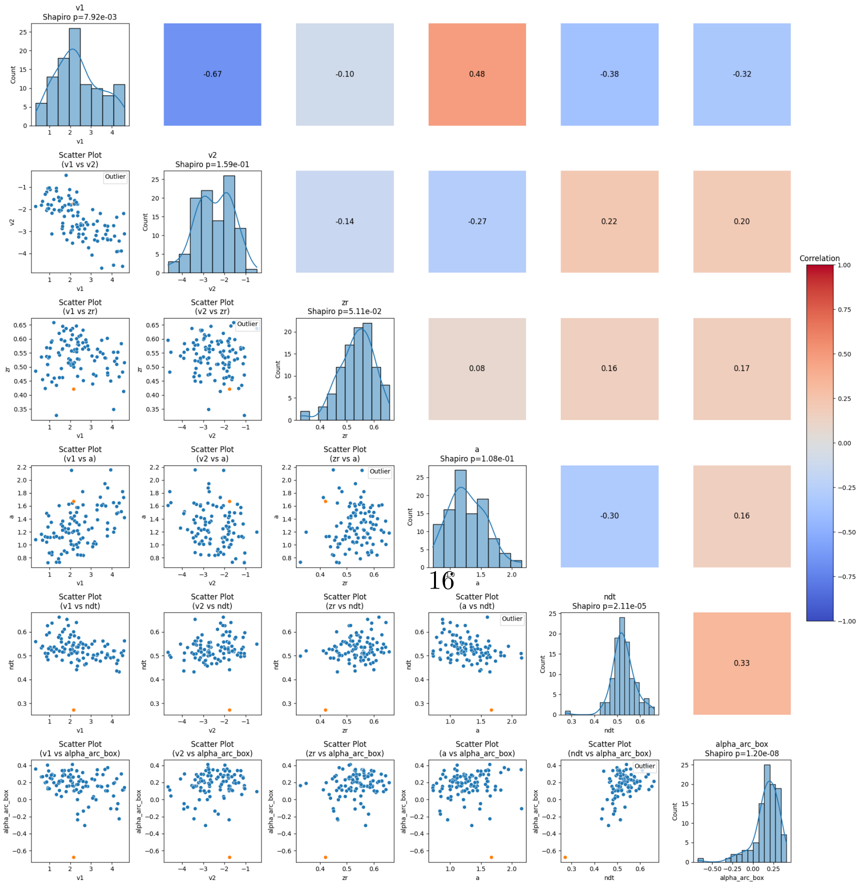

(b) RMT Session 2 Model 1

Fig. E.6: Correlation among LFM parameters for RMT Model 1

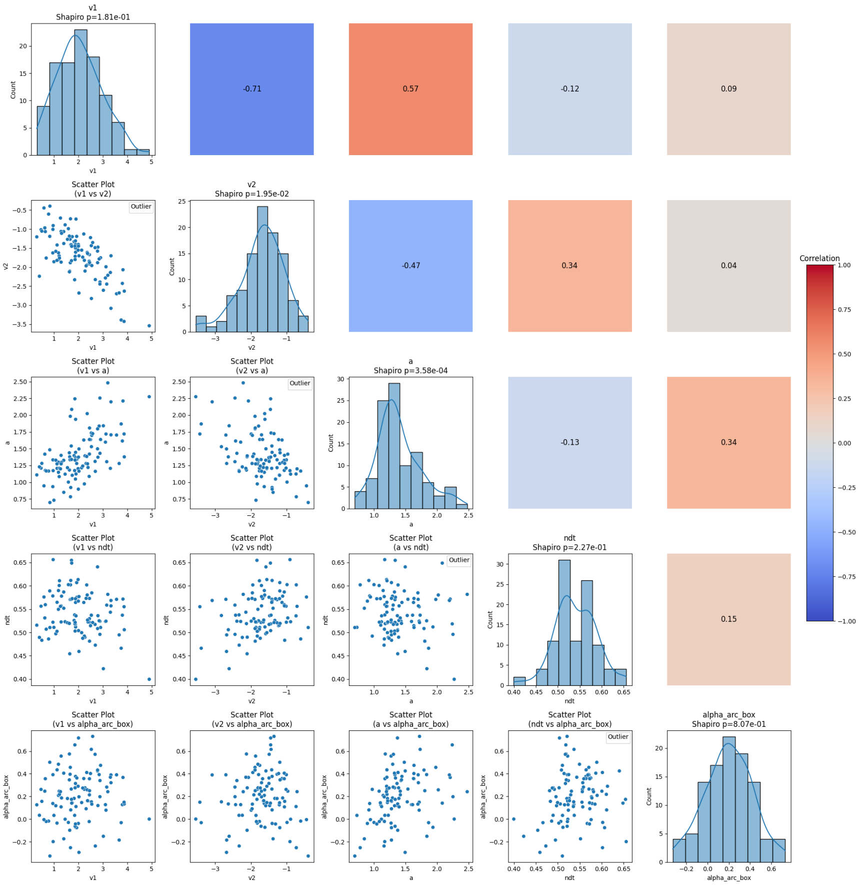

(a) RMT Session 1 Model 2

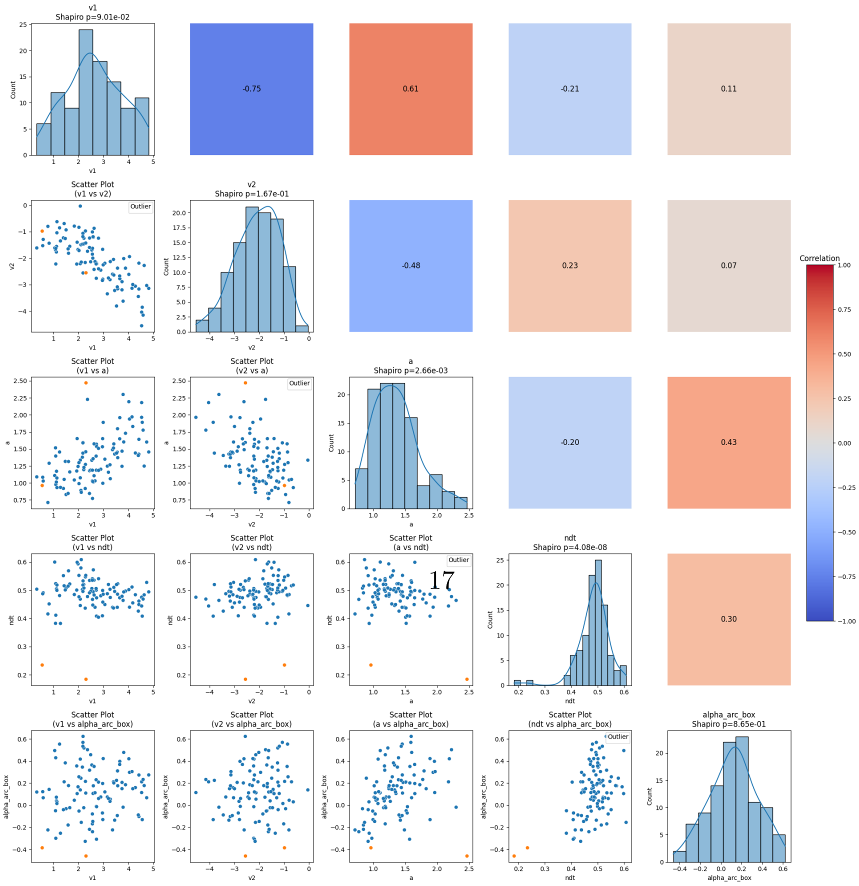

(b) RMT Session 2 Model 2

Fig. E.7: Correlation among LFM parameters for RMT Model 2

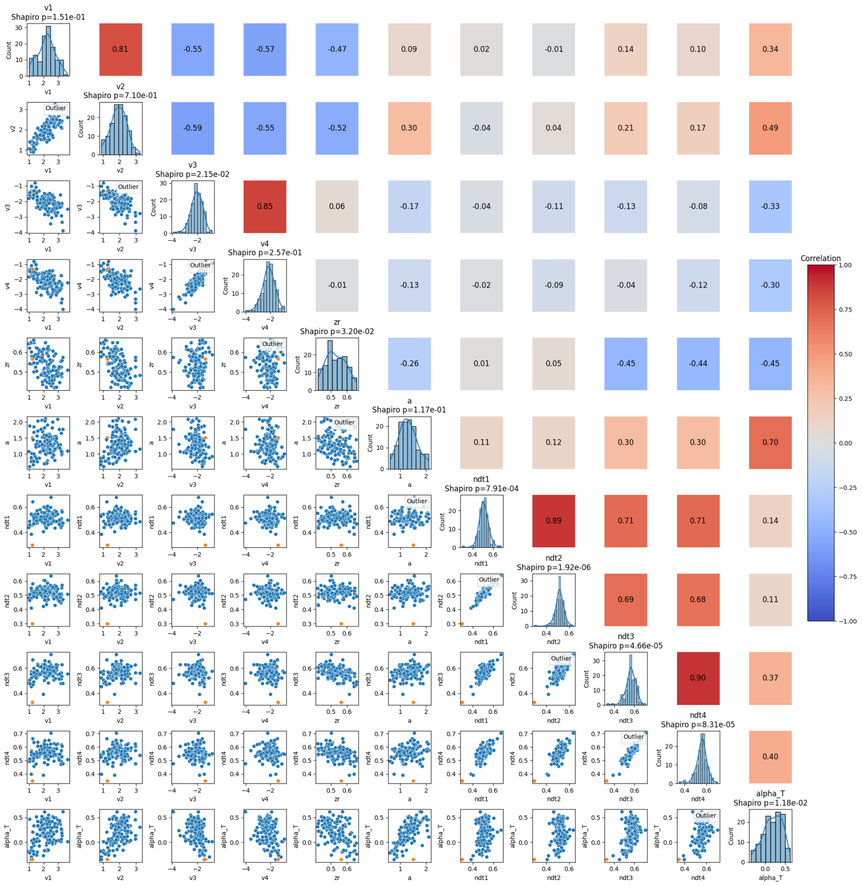

(a) APT Session 1 Model 1

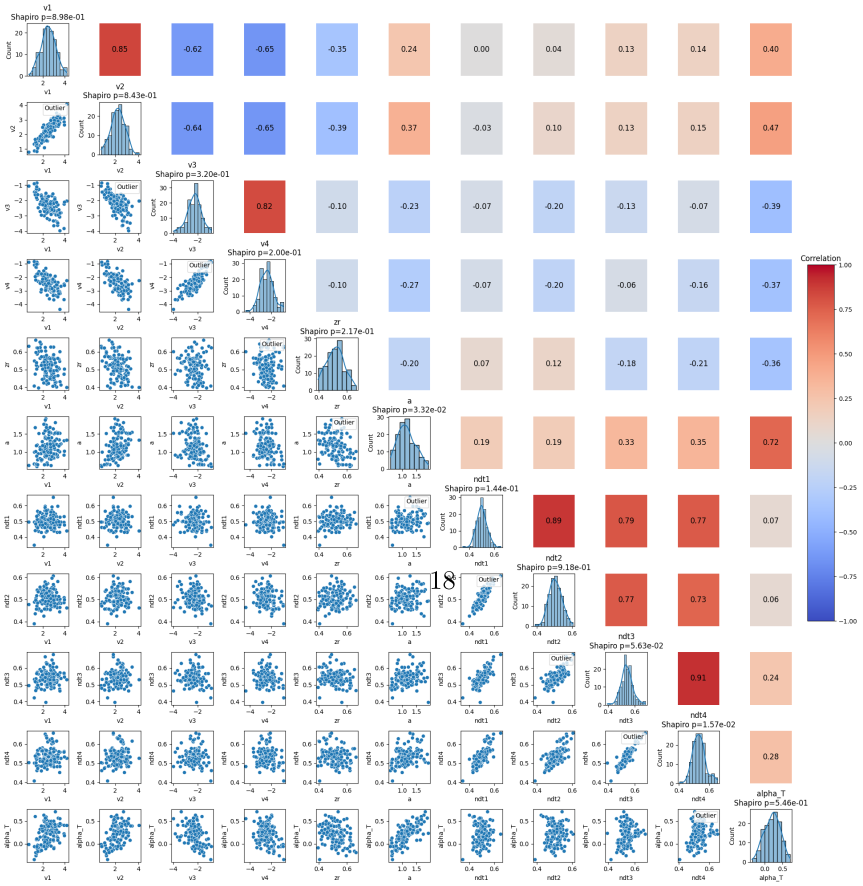

(b) APT Session 2 Model 1

Fig. E.8: Correlation among LFM parameters for APT Model 1

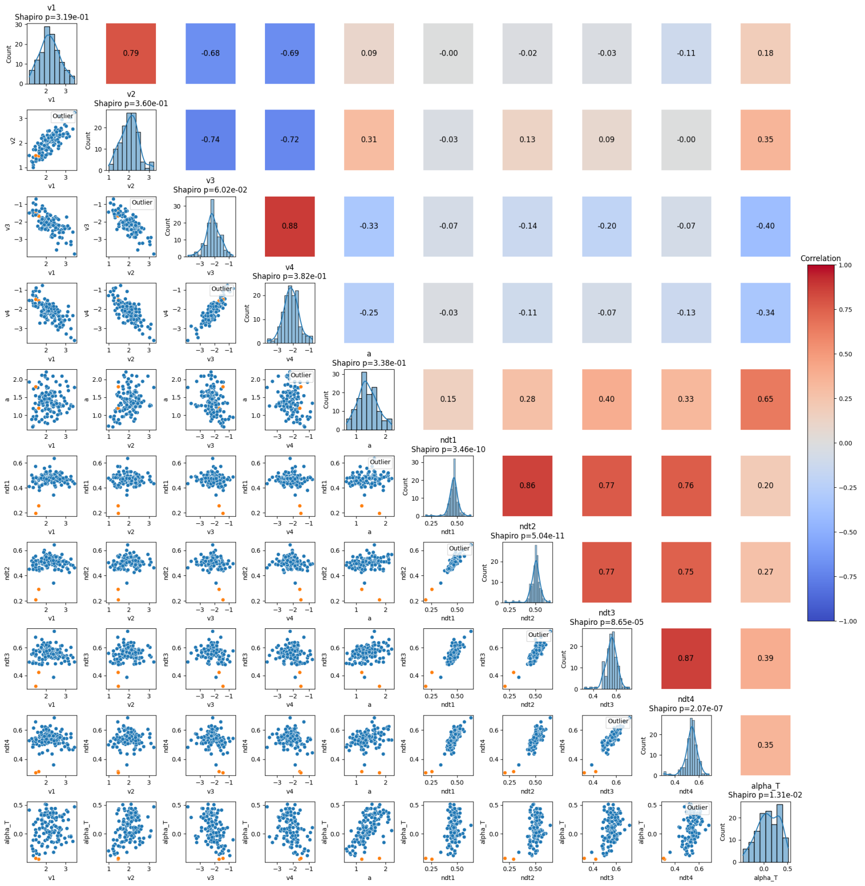

(a) APT Session 1 Model 2

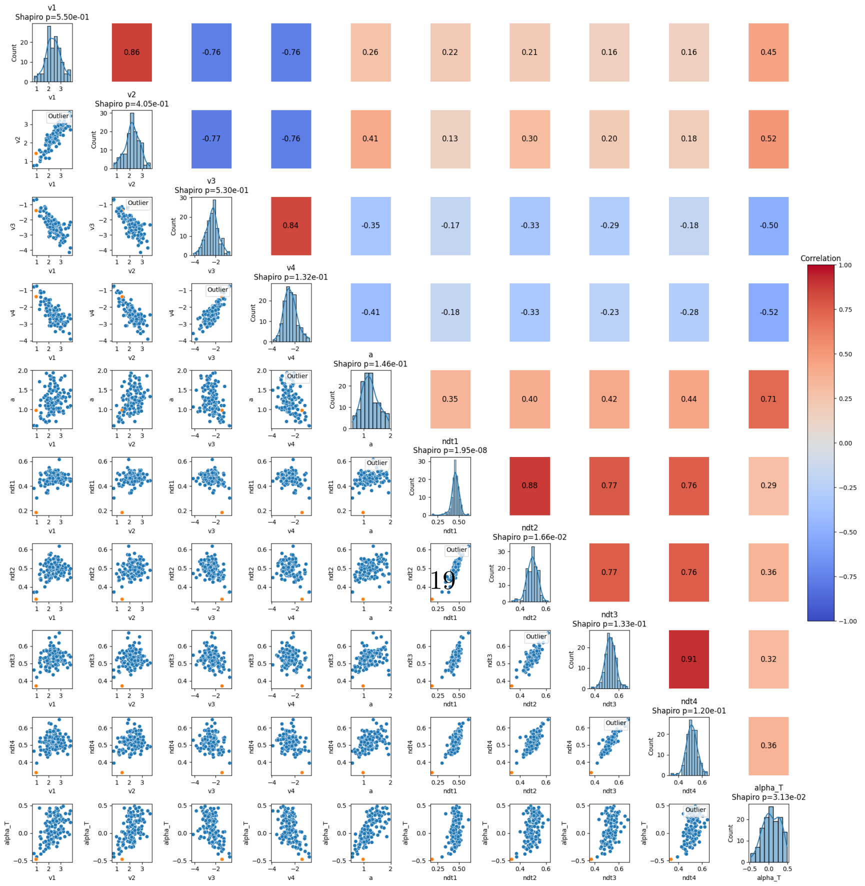

(b) APT Session 2 Model 2

Fig. E.9: Correlation among LFM parameters for APT Model 2

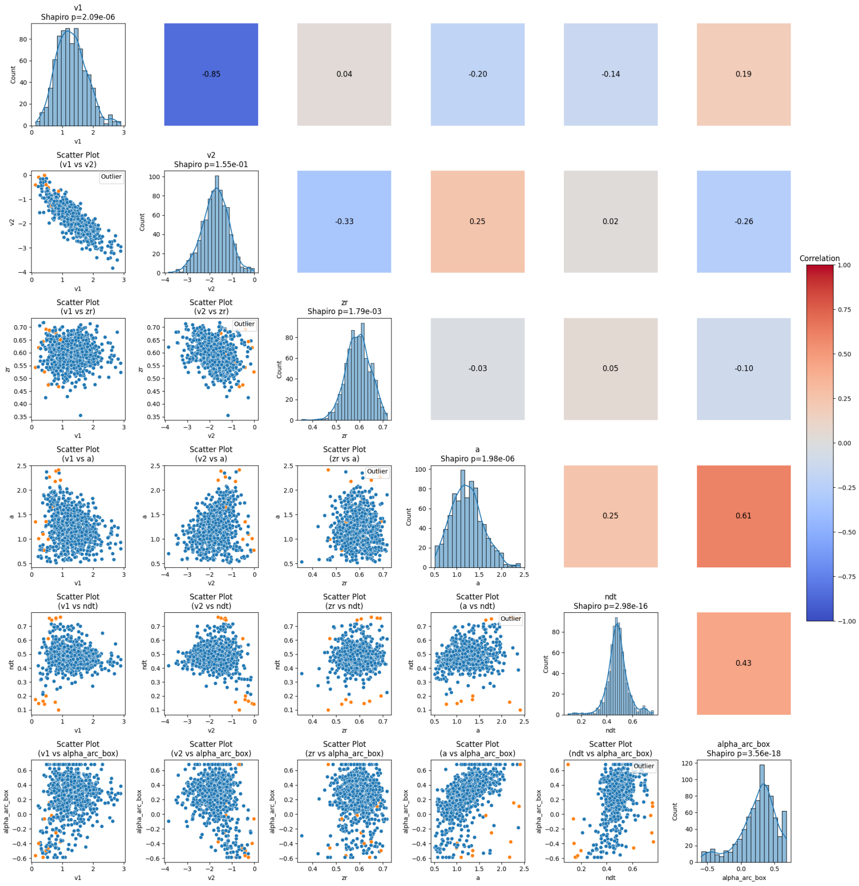

(a) ELP Session 1 Model 1

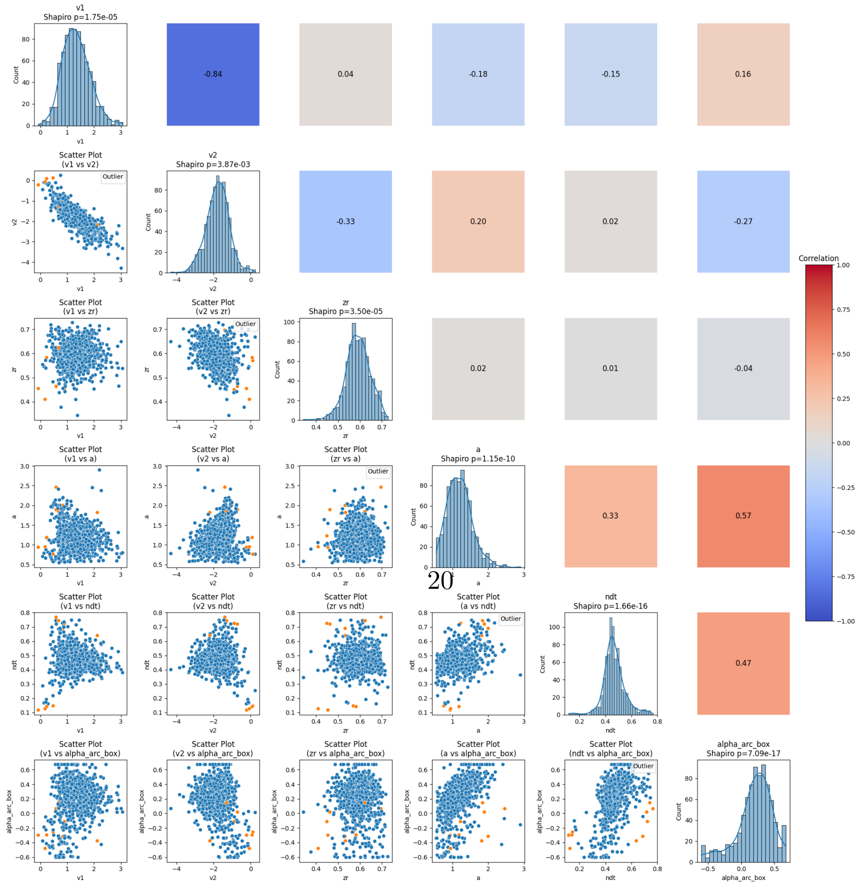

(b) ELP Session 2 Model 1

Fig. E.10: Correlation among LFM parameters for ELP Model 1

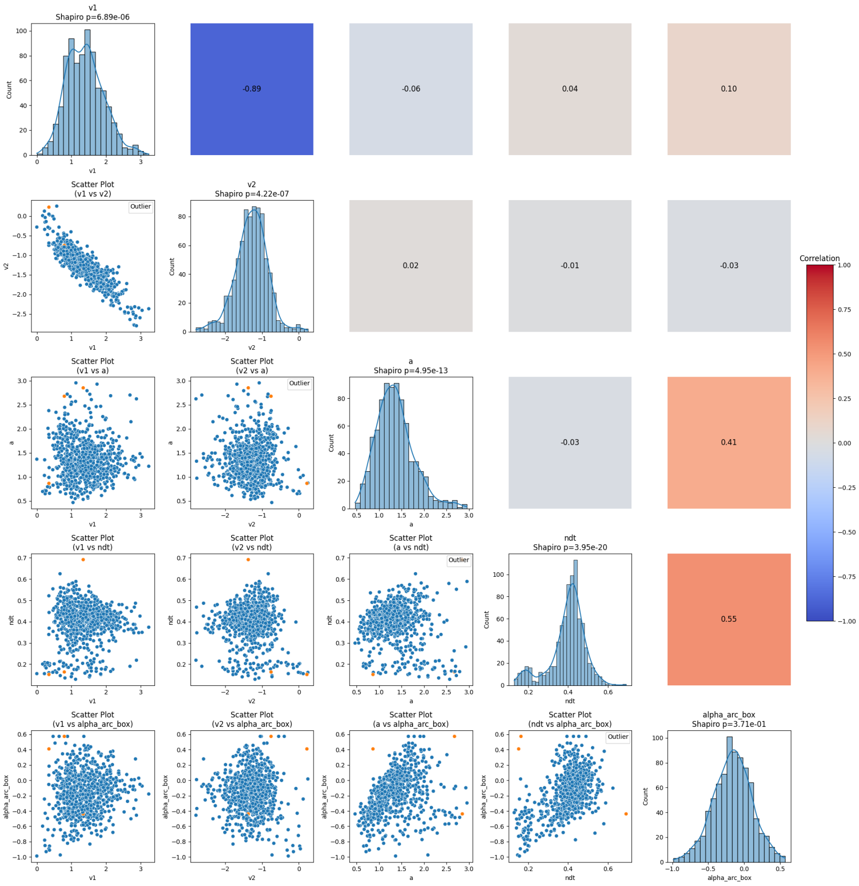

(a) ELP Session 1 Model 2

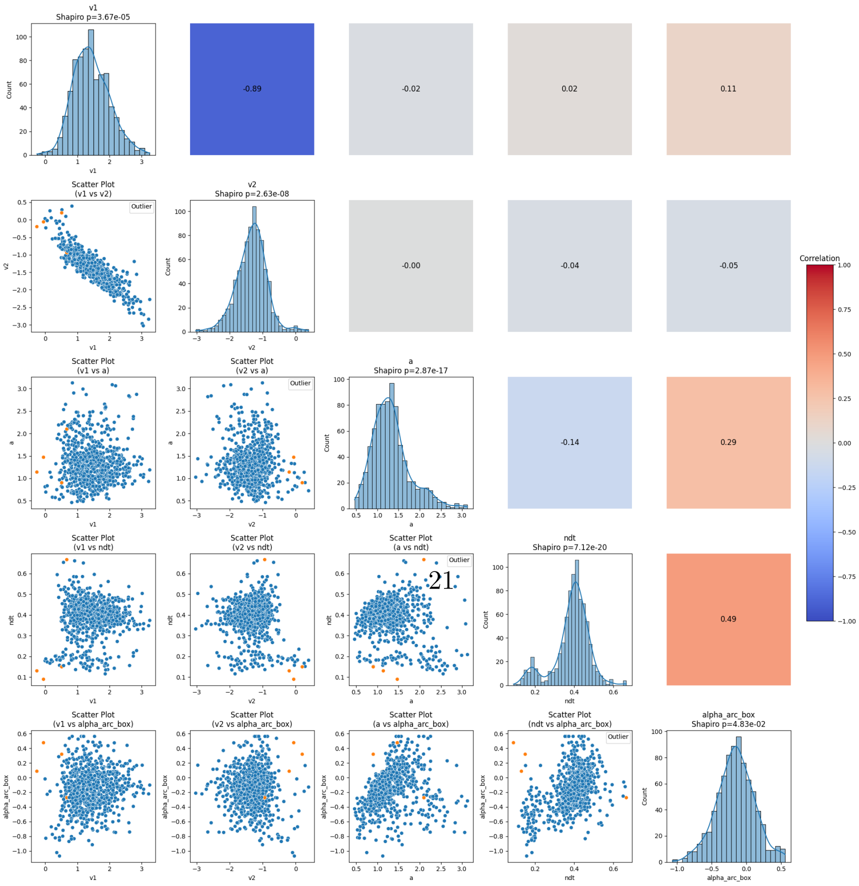

(b) ELP Session 2 Model 2

Fig. E.11: Correlation among LFM parameters for ELP Model 2

References

Nolan, J.P. (2020). *Univariate stable distributions: Models for heavy tailed data* (1st ed. ed.). Springer.
